# Linguistic neutrosophic Hamacher aggregation operators and the application in evaluating land reclamation schemes for mines

**DOI:** 10.1371/journal.pone.0206178

**Published:** 2018-11-06

**Authors:** Weizhang Liang, Guoyan Zhao, Suizhi Luo

**Affiliations:** 1 School of Resources and Safety Engineering, Central South University, Changsha, China; 2 School of Systems Engineering, National University of Defense Technology, Changsha, China; Irstea, FRANCE

## Abstract

Land reclamation has become a significant way for the improvement of ecological environment in mining areas. When selecting the optimal land reclamation scheme, LNNs (linguistic neutrosophic numbers) are suitable to describe the complex fuzzy evaluation information through linguistic truth, indeterminacy and falsity membership degrees. Furthermore, the Hamacher aggregation operators are good tools to handle multi-criteria decision making problems. Accordingly, the aim of this paper is to extend Hamacher aggregation operators with LNNs and then build a decision making framework for evaluating land reclamation schemes in mining areas. First, new operational laws of LNNs based on Hamacher t-norm and t-conorm are defined. Then, several linguistic neutrosophic Hamacher aggregation operators, including the linguistic neutrosophic Hamacher weighted mean aggregation operators and linguistic neutrosophic Hamacher hybrid weighted mean aggregation operators are developed. Meanwhile, their desirable properties are proved. Thereafter, a method for decision making with linguistic neutrosophic information based on these operators is proposed to deal with complex decision problems. At last, the validity of this method is confirmed by an illustrative example of evaluating the land reclamation schemes in mining areas. In addition, the impact of the parameter in extended Hamacher aggregation operators is discussed. The merits of the proposed method are also highlighted by comparing with other decision making methods. The results show that the proposed linguistic neutrosophic Hamacher aggregation operators have great flexibility and advantages, and can provide powerful ways for the evaluation of land reclamation schemes.

## 1. Introduction

Over years, the demand for mineral resources increases sharply due to the rapid industrial developments and global population growth [1,2]. At the same time, the large-scale exploitation of mineral resources has brought a lot of issues to the surrounding environment [3]. Specifically, a large number of land resources are polluted and destroyed [4]. In order to protect ecological environment and achieve sustainable development of mining industry, it is urgent and vital to restore such damaged lands after the completion of mining operations. Mine land reclamation is the process of restoring the land that was destroyed during the mining exploitation to some degree [5]. On account of big differences in various mining area, a specific land reclamation scheme is required for a certain mine. Thus, numbers of multi-criteria decision making methods have been adopted to assess the land reclamation schemes in mine areas, like the AHP (analytical hierarchy process) [6], linear programming model [7], TOPSIS (technique for order preference by similarity to an ideal solution) [8], limit comprehensive conditions approach [9] and fuzzy comprehensive evaluation method [10].

However, the assessment information is expressed by real numbers in all these methods mentioned above. These crisp numbers cannot describe fuzziness and uncertainty in the process of evaluation [11]. Consequently, Zadeh [12] presented the fuzzy set to deal with fuzzy and uncertain information. Since then, numerous of fuzzy decision making methods based on fuzzy set and its extensions have been developed and widely applied in various fields [13,14,15,16,17]. In order to make comprehensive appraisal of mine land reclamation, Zhou et al. [18] proposed a modified VIKOR (vlsekriterijumska optimizacija i kompromisno resenje) method with triangle fuzzy numbers. On the other hand, considering that people often use linguistic phrases such as “bad” and “good” in the process of making assessments, the concept of linguistic term sets was put forward [19]. While there are still some imperfections of the representation of linguistic term sets. There is a hypothesis that the membership degree of a certain linguistic term is one, and the indeterminacy and inconsistency cannot be depicted by linguistic term sets [20,21].

Consequently, Fang and Ye [22] proposed LNNs (linguistic neutrosophic numbers) to deal with consistent, indeterminate and inconsistent linguistic information. After that, researchers paid great attentions to LNNs and suggested many linguistic neutrosophic decision making methods. Shi and Ye [23] presented a multi-attribute group decision making method based on the cosine similarity of LNNs; Liang et al. [24] adopted the TOPSIS approach with LNNs to assess the investment risk of metallic mines; Fan et al. [25] combined LNNs with Bonferroni averaging operators to handle linguistic neutrosophic information; Liu et al. [26] advised linguistic neutrosophic power Heronian averaging operators to aggregate LNNs; Liu and You [27] developed some Hamy averaging operators with LNNs to solve group decision problems; Liang et al. [28] extended the MULTIMOORA (multi-objective optimization by ratio analysis plus the full multiplicative form) method under linguistic neutrosophic circumstance.

The information aggregation operators have been always regarded as important and interesting topics in decision making areas. Garg and Nancy [29,30] defined some novel aggregation operators for neutrosophic numbers; Liu and Qin [31] extended the Maclaurin symmetric mean operators with LIFNs (linguistic intuitionistic fuzzy numbers) and discussed their application in multi-criteria decision making fields; Garg and Nancy [32] developed the priority aggregation operators to aggregate linguistic single-valued neutrosophic information; Garg and Kumar [33] proposed several aggregation operators of LIFNs to overcome the shortcomings of the existing operators; Garg and Nancy [34] discussed the prioritized Muirhead mean aggregation operator under neutrosophic set environment; Liu et al. [35] recommended the linguistic intuitionistic fuzzy partitioned Heronian means to tackle decision making issues.

These mentioned-above operators are all on the basis of the same type of operations, namely, the algebraic operational rules. Generally, another useful type of operations, the Hamacher t-norm and t-conorm, can be also utilized to model the intersection and union of various fuzzy sets [36]. A great number of extended Hamacher aggregation operators based on different fuzzy number situations have been developed [37,38,39]. Recently, Garg et al. [40] extended Hamacher t-norm and t-conorm with interval intuitionistic fuzzy numbers to deal with multi-criteria decision making problems; Gao et al. [41] presented some Hamacher prioritized operators for aggregating dual hesitant bipolar fuzzy numbers; Wu et al. [42] combined Hamacher aggregation operators with single-valued neutrosophic 2-tuple linguistic numbers; Li et al. [43] defined the multivalued neutrosophic linguistic normalized weighted Bonferroni mean Hamacher operators; Tang and Meng [44] discussed the Hamacher aggregation operators under linguistic intuitionistic fuzzy environment. Nevertheless, the Hamacher aggregation operators have not been extended to LNNs so far.

Overall, the motivations of this paper can be summarized as follows.

1. The evaluation of land reclamation schemes is a complicated decision making problem with many uncertain factors. Nevertheless, evaluation information is just described by real numbers in the existing reclamation schemes selection methods, which may lead to information loss or distortion. Thus, for land reclamation schemes selection, finding a pertinent way to describe fuzzy or uncertain evaluation information is essential.
2. As a popular set, LNNs show great performance in expressing consistent, indeterminate and inconsistent evaluation information. Hence, LNNs may be a good choice to depict fuzzy assessment values for the selection of land reclamation schemes.
3. Considering that using information aggregation operators is a simple but powerful way to solve majority of decision making problems, this paper tends to define new aggregation operators under linguistic neutrosophic environment to address complex land reclamation schemes selection issues.

Consequently, a linguistic neutrosophic decision making method based on Hamacher aggregation operators is proposed to select the optimal land reclamation scheme in this paper. Compared with other methods, the advantages of the proposed work are three-fold. First, the proposed approach expresses decision making information using LNNs, which can describe the linguistic evaluation information more adequately. Second, the Hamacher aggregation operators with one parameter are more flexible and powerful in the information fusion and decision making process. Third, exploring some Hamacher aggregation operators for LNNs and applying them to model practical decision making problems are necessary and meaningful. The combination of LNNs and Hamacher aggregation operators can be much benefit for coping with complex decision making problems, like the selection of land reclamation schemes.

The novelties and contributions of this paper are listed as follows.

1. New operational rules, the Hamacher operational rules of LNNs are proposed. Based on them, the Hamacher aggregation operators are extended to LNNs and major properties are justified. They are general and flexible family of continuous triangular norms, and can be regarded as the generalization of Algebraic and Einstein t-norm and t-conorm.
2. A decision making model based on the linguistic neutrosophic Hamacher aggregation operators is constructed to tackle the problem of assessing land reclamation schemes. Like algebraic operators, Hamacher aggregation operators have the function of smooth approximation as well. Moreover, LNNs are good at expressing consistent and inconsistent qualitative information.
3. In the proposed method, not only the weights of criteria, but also the weights of ordered positions are under consideration.
4. The evaluation criteria of land reclamation schemes are recognized, and a case is studied to explain the calculation process and verify the practicality of our method. Furthermore, the features and strengths of the proposed method are demonstrated through sensitivity and comparison analyses.

The rest part of this paper is allocated as follows. Section 2 recalls some basic concepts and notations, such as the linguistic term set, LNNs, t-norm and t-conorm. Besides, the linguistic neutrosophic Hamacher operational laws are firstly defined. In Section 3, two main types of linguistic neutrosophic Hamacher operators are presented and some desirable properties are discussed. Thereafter, a method for decision making with linguistic neutrosophic information is recommended in Section 4. Section 5 gives an illustrative example of appraising land reclamation schemes in mine areas after the evaluation criteria system is constructed. In Section 6, the sensitivity and comparison analyses are respectively undertaken. Finally, concluding remarks are provided.

## 2. Preliminaries

In this section, general concepts of LNNs, t-norm and t-conorm are reviewed, and then new operational laws of LNNs based on Hamacher t-norm and t-conorm are introduced.

### 2.1 General concepts

**Definition 1**. [19] Suppose there are a group of linguistic variables *t*_*i*_ (*i* = 0, 1,⋯,2*h*). For all *i*, *j* = 0, 1,⋯,2*h*, the followings are true: (1) When *i* < *j*, then *t*_*i*_ < *t*_*j*_; (2) when *i* = *j*, then *t*_*i*_ = *t*_*j*_; (3) when *i* > *j*, then *t*_*i*_ > *t*_*j*_. And the operational rules of linguistic variables are: *t*_*i*_ ⊕ *t*_*j*_ = *t*_*i*+*j*_ and *r* ⋅ *t*_*i*_ = *t*_*ri*_ (*r* ≥ 0). When a collection of linguistic values exist, a discrete linguistic term set is denoted as T* = {*t*_*i*_|*i* = 0, 1,⋯,2*h*} (*h* > 0), and a continuous linguistic term set is expressed as *T* = {*t*_*i*_ | *i* ∈ [0,2*s*]} (*s* > 0).

**Definition 2**. [22] A LNN (linguistic neutrosophic number) is denoted as $a=(t_{T_{a}},t_{I_{a}},t_{F_{a}})$, where $t_{T_{a}}$, $t_{I_{a}}$ and $t_{F_{a}}$ are three independent linguistic terms, which respectively represent true, hesitant and false membership degrees.

**Definition 3**. [22] If *T* = {*t*_*i*_ | *i* ∈ [0,2*s*]} is a continuous linguistic term set and $a=(t_{T_{a}},t_{I_{a}},t_{F_{a}})$ is a LNN, then the score function of *a* is *U*(*a*) = (4*s* + *T*_*a*_ − *I*_*a*_ − *F*_*a*_)/6*s*, and the accuracy function of *a* is *V*(*a*) = (*T*_*a*_ − *F*_*a*_)/2*s*.

**Definition 4**. [45] Let $b=(l_{T_{b}},l_{I_{b}},l_{F_{b}})$ and $c=(l_{T_{c}},l_{I_{c}},l_{F_{c}})$ two LNNs, their order relationship can be described as follows:

1. if *U*(*b*) > *U*(*c*), then *b* ≻ *c*;
2. if *U*(*b*) = *U*(*c*), then $\{\begin{array}{c} V(b)>V(c)\Rightarrow b≻c\\ V(b)=V(c)\Rightarrow b≃c \end{array}$.

**Definition 5**. [46]. The t-norm and t-conorm are the most general families of binary functions, which contain all types of the particular operators. Three special cases of t-norm and t-conorm are itemized as follows:

1. Algebraic t-norm and t-conorm:
$$T^{A}(x,y)=x\times y,$$ (1)
$$S^{A}(x,y)=x+y-x\times y(0\leq x,y\leq 1).$$ (2)
2. Einstein t-norm and t-conorm:
$$T^{E}(x,y)=\frac{x\times y}{1+(1-x)\times (1-y)},$$ (3)
$$S^{E}(x,y)=\frac{x+y}{1+x\times y}(0\leq x,y\leq 1).$$ (4)
3. Hamacher t-norm and t-conorm:
$$T^{H\lambda }(x,y)=\frac{x\times y}{\lambda +(1-\lambda )\times (x+y-xy)},$$ (5)
$$S^{H\lambda }(x,y)=\frac{x+y-xy-(1-\lambda )xy}{1-(1-\lambda )xy}(0\leq x,y\leq 1,\lambda >0).$$ (6)

Note that when *λ* = 1, the Hamacher t-norm and t-conorm are respectively degenerated into Algebraic t-norm and t-conorm; and when *λ* = 2, the Hamacher t-norm and t-conorm are respectively degenerated into Einstein t-norm and t-conorm.

### 2.2 Operational laws of linguistic neutrosophic numbers based on Hamacher t-norm and t-conorm

**Definition 6**. Given a continuous linguistic term set *T* = {*t*_*i*_ | *i* ∈ [0,2*s*]} and two arbitrary LNNs $b=(t_{T_{b}},t_{I_{b}},t_{F_{b}})$ and $c=(t_{T_{c}},t_{I_{c}},t_{F_{c}})$. Assume a conversion function is $g(t_{i})=\frac{i}{2s}$ and the corresponding converse function is *g*^−1^(*i*) = *t*_2*s*×*i*_, then the operational laws of them based on Hamacher t-norm and t-conorm are defined as follows:

$$\begin{array}{ll} b⊕c=\left(g^{-1}\left(S^{H\lambda }\left(g(t_{T_{b}}),g(t_{T_{c}})\right)\right),g^{-1}\left(T^{H\lambda }\left(g(t_{I_{b}}),g(t_{I_{c}})\right)\right),g^{-1}\left(T^{H\lambda }\left(g(t_{F_{b}}),g(t_{F_{c}})\right)\right)\right)\\ \begin{array}{l} =(g^{-1}\left(\frac{g(t_{T_{b}})+g(t_{T_{c}})-g(t_{T_{b}})g(t_{T_{c}})-(1-\lambda )g(t_{T_{b}})g(t_{T_{c}})}{1-(1-\lambda )g(t_{T_{b}})g(t_{T_{c}})}\right),g^{-1}\left(\frac{g(t_{I_{b}})\times g(t_{I_{c}})}{\lambda +(1-\lambda )\times (g(t_{I_{b}})+g(t_{I_{c}})-g(t_{I_{b}})g(t_{I_{c}}))}\right),\\ g^{-1}\left(\frac{g(t_{F_{b}})\times g(t_{F_{c}})}{\lambda +(1-\lambda )\times (g(t_{F_{b}})+g(t_{F_{c}})-g(t_{F_{b}})g(t_{F_{c}}))}\right)) \end{array} & ; \end{array}$$ (7)

$$\begin{array}{l} b⊗c=\left(g^{-1}\left(T^{H\lambda }\left(g(t_{T_{b}}),g(t_{T_{c}})\right)\right),g^{-1}\left(S^{H\lambda }\left(g(t_{I_{b}}),g(t_{I_{c}})\right)\right),g^{-1}\left(S^{H\lambda }\left(g(t_{F_{b}}),g(t_{F_{c}})\right)\right)\right)\\ \begin{array}{l} =(g^{-1}\left(\frac{g(t_{T_{b}})\times g(t_{T_{c}})}{\lambda +(1-\lambda )\times (g(t_{T_{b}})+g(t_{T_{c}})-g(t_{T_{b}})g(t_{T_{c}}))}\right),g^{-1}\left(\frac{g(t_{I_{b}})+g(t_{I_{c}})-g(t_{I_{b}})g(t_{I_{c}})-(1-\lambda )g(t_{I_{b}})g(t_{I_{c}})}{1-(1-\lambda )g(t_{I_{b}})g(t_{I_{c}})}\right),\\ g^{-1}\left(\frac{g(t_{F_{b}})+g(t_{F_{c}})-g(t_{F_{b}})g(t_{F_{c}})-(1-\lambda )g(t_{F_{b}})g(t_{F_{c}})}{1-(1-\lambda )g(t_{F_{b}})g(t_{F_{c}})}\right)) \end{array} \end{array};$$ (8)

$$\delta b=\begin{array}{l} (g^{-1}\left(\frac{{\left(1+(\lambda -1)(g(t_{T_{b}}))\right)}^{\delta }-{\left(1-g(t_{T_{b}})\right)}^{\delta }}{{\left(1+(\lambda -1)(g(t_{T_{b}}))\right)}^{\delta }+(\lambda -1){\left(1-g(t_{T_{b}})\right)}^{\delta }}\right),g^{-1}\left(\frac{\lambda {\left(g(t_{I_{b}})\right)}^{\delta }}{{\left(1+(\lambda -1)\left(1-g(t_{I_{b}})\right)\right)}^{\delta }+(\lambda -1){\left(g(t_{I_{b}})\right)}^{\delta }}\right),\\ g^{-1}\left(\frac{\lambda {\left(g(t_{F_{b}})\right)}^{\delta }}{{\left(1+(\lambda -1)(1-g(t_{F_{b}}))\right)}^{\delta }+(\lambda -1){\left(g(t_{F_{b}})\right)}^{\delta }}\right)) \end{array};$$ (9)

$$b^{\delta }=\begin{array}{l} (g^{-1}\left(\frac{\lambda {\left(g(t_{T_{b}})\right)}^{\delta }}{{\left(1+(\lambda -1)(1-g(t_{T_{b}}))\right)}^{\delta }+(\lambda -1){\left(g(t_{T_{b}})\right)}^{\delta }}\right),g^{-1}\left(\frac{{\left(1+(\lambda -1)(g(t_{I_{b}}))\right)}^{\delta }-{\left(1-g(t_{I_{b}})\right)}^{\delta }}{{\left(1+(\lambda -1)(g(t_{I_{b}}))\right)}^{\delta }+(\lambda -1){\left(1-g(t_{I_{b}})\right)}^{\delta }}\right),\\ \;g^{-1}\left(\frac{{\left(1+(\lambda -1)(g(t_{F_{b}}))\right)}^{\delta }-{\left(1-g(t_{F_{b}})\right)}^{\delta }}{{\left(1+(\lambda -1)(g(t_{F_{b}}))\right)}^{\delta }+(\lambda -1){\left(1-g(t_{F_{b}})\right)}^{\delta }}\right)) \end{array}.$$ (10)

Note that when *λ* = 1, the operational laws of LNNs based on Hamacher t-norm and t-conorm defined in Definition 4 are reduced linguistic neutrosophic Algebraic operational laws as follows:

$$b{⊕}_{A}c=\left(g^{-1}\left(g(t_{T_{b}})+g(t_{T_{c}})-g(t_{T_{b}})g(t_{T_{c}})\right),g^{-1}\left(g(t_{I_{b}})\times g(t_{I_{c}})\right),g^{-1}\left(g(t_{F_{b}})\times g(t_{F_{c}})\right)\right);$$ (11)

$$b{⊗}_{A}c=\left(g^{-1}\left(g(t_{T_{b}})\times g(t_{T_{c}})\right),g^{-1}\left(g(t_{I_{b}})+g(t_{I_{c}})-g(t_{I_{b}})g(t_{I_{c}})\right),g^{-1}\left(g(t_{F_{b}})+g(t_{F_{c}})-g(t_{F_{b}})g(t_{F_{c}})\right)\right);$$ (12)

$$\delta b=\left(g^{-1}\left(1-{\left(1-g(t_{T_{b}})\right)}^{\delta }\right),g^{-1}\left({\left(g(t_{I_{b}})\right)}^{\delta }\right),g^{-1}\left({\left(g(t_{F_{b}})\right)}^{\delta }\right)\right);$$ (13)

$$b^{\delta }=\left(g^{-1}\left({\left(g(t_{T_{b}})\right)}^{\delta }\right),g^{-1}\left(1-{\left(1-g(t_{I_{b}})\right)}^{\delta }\right),g^{-1}\left(1-{\left(1-g(t_{F_{b}})\right)}^{\delta }\right)\right).$$ (14)

When *λ* = 2, the operational laws of LNNs based on Hamacher t-norm and t-conorm defined in Definition 4 are reduced linguistic neutrosophic Einstein operational laws as follows:

$$b{⊕}_{E}c=\left(g^{-1}\left(\frac{g(t_{T_{b}})+g(t_{T_{c}})}{1+g(t_{T_{b}})g(t_{T_{c}})}\right),g^{-1}\left(\frac{g(t_{I_{b}})\times g(t_{I_{c}})}{2-g(t_{I_{b}})-g(t_{I_{c}})+g(t_{I_{b}})g(t_{I_{c}})}\right),g^{-1}\left(\frac{g(t_{F_{b}})\times g(t_{F_{c}})}{2-g(t_{F_{b}})-g(t_{F_{c}})+g(t_{F_{b}})g(t_{F_{c}})}\right)\right);$$ (15)

$$b{⊗}_{E}c=\left(g^{-1}\left(\frac{g(t_{T_{b}})\times g(t_{T_{c}})}{2-g(t_{T_{b}})-g(t_{T_{c}})+g(t_{T_{b}})g(t_{T_{c}})}\right),g^{-1}\left(\frac{g(t_{I_{b}})+g(t_{I_{c}})}{1+g(t_{I_{b}})g(t_{I_{c}})}\right),g^{-1}\left(\frac{g(t_{F_{b}})+g(t_{F_{c}})}{1+g(t_{F_{b}})g(t_{F_{c}})}\right)\right);$$ (16)

$$\delta b=\left(g^{-1}\left(\frac{{\left(1+g(t_{T_{b}})\right)}^{\delta }-{\left(1-g(t_{T_{b}})\right)}^{\delta }}{{\left(1+g(t_{T_{b}})\right)}^{\delta }+{\left(1-g(t_{T_{b}})\right)}^{\delta }}\right),g^{-1}\left(\frac{2{\left(g(t_{I_{b}})\right)}^{\delta }}{{\left(1+(1-g(t_{I_{b}}))\right)}^{\delta }+{\left(g(t_{F_{b}})\right)}^{\delta }}\right),g^{-1}\left(\frac{2{\left(g(t_{F_{b}})\right)}^{\delta }}{{\left(1+(1-g(t_{F_{b}}))\right)}^{\delta }+{\left(g(t_{F_{b}})\right)}^{\delta }}\right)\right);$$ (17)

$$b^{\delta }=\left(g^{-1}\left(\frac{2{\left(g(t_{T_{b}})\right)}^{\delta }}{{\left(1+(1-g(t_{T_{b}}))\right)}^{\delta }+{\left(g(t_{T_{b}})\right)}^{\delta }}\right),g^{-1}\left(\frac{{\left(1+g(t_{I_{b}})\right)}^{\delta }-{\left(1-g(t_{I_{b}})\right)}^{\delta }}{{\left(1+g(t_{I_{b}})\right)}^{\delta }+{\left(1-g(t_{I_{b}})\right)}^{\delta }}\right),g^{-1}\left(\frac{{\left(1+g(t_{F_{b}})\right)}^{\delta }-{\left(1-g(t_{F_{b}})\right)}^{\delta }}{{\left(1+g(t_{F_{b}})\right)}^{\delta }+{\left(1-g(t_{F_{b}})\right)}^{\delta }}\right)\right).$$ (18)

## 3. Linguistic neutrosophic Hamacher aggregation operators

In this section, some linguistic neutrosophic Hamacher aggregation operators are proposed based on operational laws of LNNs defined in subsection 2.2.

### 3.1 Linguistic neutrosophic Hamacher weighted mean operators

**Definition 7**. If $d_{i}=(t_{T_{i}},t_{I_{i}},t_{F_{i}})$ is a collection of LNNs on the linguistic term set *T* = {*t*_*i*_ | *i* ∈ [0,2*s*]}, and the weight vector is *w* = (*w*_1_, *w*_2_, ⋯, *w*_*n*_) where *w*_*i*_ ∈ [0,1] (*i* = 1,2,⋯,*n*) and *w*_1_ + *w*_2_ +⋯+*w*_*n*_ = 1. Then the linguistic neutrosophic Hamacher weighted arithmetic mean (*LNHWAM*^*λ*^) operator is

$$LNHWAM^{\lambda }(d_{1},d_{2},\cdots ,d_{n})={⊕}_{i=1}^{n}(w_{i}d_{i})=w_{1}d_{1}⊕w_{2}d_{2}⊕\cdots ⊕w_{n}d_{n}.$$ (19)

**Theorem 1**. Let *T* = {*t*_*i*_ | *i* ∈ [0,2*s*]} be a continuous linguistic term set, and $d_{i}=(t_{T_{i}},t_{I_{i}},t_{F_{i}})$ is a set of LNNs, then the aggregated value by using *LNHWAM*^*λ*^ operator is still a LNN, which is shown as follows:

$$\begin{array}{l} LNHWAM^{\lambda }(d_{1},d_{2},\cdots ,d_{n})=\\ (g^{-1}\left(\frac{\prod_{i=1}^{n}{\left(1+(\lambda -1)(g(t_{T_{i}}))\right)}^{w_{i}}-\prod_{i=1}^{n}{\left(1-g(t_{T_{i}})\right)}^{w_{i}}}{\prod_{i=1}^{n}{\left(1+(\lambda -1)(g(t_{T_{i}}))\right)}^{w_{i}}+(\lambda -1)\prod_{i=1}^{n}{\left(1-g(t_{T_{i}})\right)}^{w_{i}}}\right),g^{-1}\left(\frac{\lambda \prod_{i=1}^{n}{\left(g(t_{I_{i}})\right)}^{w_{i}}}{\prod_{i=1}^{n}{\left(1+(\lambda -1)\left(1-g(t_{I_{i}})\right)\right)}^{w_{i}}+(\lambda -1)\prod_{i=1}^{n}{\left(g(t_{I_{i}})\right)}^{w_{i}}}\right),\\ g^{-1}\left(\frac{\lambda \prod_{i=1}^{n}{\left(g(t_{F_{i}})\right)}^{w_{i}}}{\prod_{i=1}^{n}{\left(1+(\lambda -1)\left(1-g(t_{F_{i}})\right)\right)}^{w_{i}}+(\lambda -1)\prod_{i=1}^{n}{\left(g(t_{F_{i}})\right)}^{w_{i}}}\right)) \end{array}.$$ (20)

The proof of this theorem is provided in Appendix A.

**Definition 8**. If $d_{i}=(t_{T_{i}},t_{I_{i}},t_{F_{i}})$ is a collection of LNNs on the linguistic term set *T* = {*t*_*i*_ | *i* ∈ [0,2*s*]}, and the weight vector is *w* = (*w*_1_, *w*_2_, ⋯, *w*_*n*_) where *w*_*i*_ ∈ [0,1] (*i* = 1,2,⋯,*n*) and *w*_1_ + *w*_2_ +⋯+*w*_*n*_ = 1. Then the linguistic neutrosophic Hamacher weighted geometric mean (*LNHWAM*^*λ*^) operator is

$$LNHWGM^{\lambda }(d_{1},d_{2},\cdots ,d_{n})={⊗}_{i=1}^{n}{\left(d_{i}\right)}^{w_{i}}={\left(d_{1}\right)}^{w_{1}}⊗{\left(d_{2}\right)}^{w_{2}}⊗\cdots ⊗{\left(d_{n}\right)}^{w_{n}}.$$ (21)

**Theorem 2**. Let *T* = {*t*_*i*_ | *i* ∈ [0,2*s*]} be a continuous linguistic term set, and $d_{i}=(t_{T_{i}},t_{I_{i}},t_{F_{i}})$ is a set of LNNs, then the aggregated value by using the *LNHWAM*^*λ*^ operator is still a LNN, which is shown as follows:

$$\begin{array}{l} LNHWGM^{\lambda }(d_{1},d_{2},\cdots ,d_{n})=\\ (g^{-1}\left(\frac{\lambda \prod_{i=1}^{n}{\left(g(t_{T_{i}})\right)}^{w_{i}}}{\prod_{i=1}^{n}{\left(1+(\lambda -1)\left(1-g(t_{T_{i}})\right)\right)}^{w_{i}}+(\lambda -1)\prod_{i=1}^{n}{\left(g(t_{T_{i}})\right)}^{w_{i}}}\right),g^{-1}(\frac{\prod_{i=1}^{n}{\left(1+(\lambda -1)(g(t_{I_{i}}))\right)}^{w_{i}}-\prod_{i=1}^{n}{\left(1-g(t_{I_{i}})\right)}^{w_{i}}}{\prod_{i=1}^{n}{\left(1+(\lambda -1)(g(t_{I_{i}}))\right)}^{w_{i}}+(\lambda -1)\prod_{i=1}^{n}{\left(1-g(t_{I_{i}})\right)}^{w_{i}}}),\\ g^{-1}\left(\frac{\prod_{i=1}^{n}{\left(1+(\lambda -1)(g(t_{F_{i}}))\right)}^{w_{i}}-\prod_{i=1}^{n}{\left(1-g(t_{F_{i}})\right)}^{w_{i}}}{\prod_{i=1}^{n}{\left(1+(\lambda -1)(g(t_{F_{i}}))\right)}^{w_{i}}+(\lambda -1)\prod_{i=1}^{n}{\left(1-g(t_{F_{i}})\right)}^{w_{i}}}\right)) \end{array}.$$ (22)

**Proof**. Since the proof of Theorem 2 is similar to that of Theorem 1, it is omitted to save space.

Note that when *λ* = 1, the *LNHWAM*^*λ*^ operator is degenerated to the linguistic neutrosophic Algebraic weighted arithmetic mean (*LNHWGM*^1^) operator as follows:

$$LNAWAM^{1}(d_{1},d_{2},\cdots ,d_{n})=\left(g^{-1}\left(1-\prod_{i=1}^{n}{\left(1-g(t_{T_{i}})\right)}^{w_{i}}\right),g^{-1}\left(\prod_{i=1}^{n}{\left(g(t_{I_{i}})\right)}^{w_{i}}\right),g^{-1}\left(\prod_{i=1}^{n}{\left(g(t_{F_{i}})\right)}^{w_{i}}\right)\right);$$ (23)

and the *LNHWGM*^*λ*^ operator is degenerated to the linguistic neutrosophic Algebraic weighted geometric mean (*LNAWGM*^1^) operator as follows:

$$LNAWGM^{1}(d_{1},d_{2},\cdots ,d_{n})=\left(g^{-1}\left(\prod_{i=1}^{n}{\left(g(t_{T_{i}})\right)}^{w_{i}}\right),g^{-1}\left(1-\prod_{i=1}^{n}{\left(1-g(t_{I_{i}})\right)}^{w_{i}}\right),g^{-1}\left(1-\prod_{i=1}^{n}{\left(1-g(t_{F_{i}})\right)}^{w_{i}}\right)\right).$$ (24)

When *λ* = 2, the *LNHWAM*^*λ*^ operator is degenerated to the linguistic neutrosophic Einstein weighted arithmetic mean (*LNEWAM*^2^) operator as follows:

$$\begin{array}{l} LNEWAM^{2}(d_{1},d_{2},\cdots ,d_{n})=\\ \left(g^{-1}\left(\frac{\prod_{i=1}^{n}{\left(1+g(t_{T_{i}})\right)}^{w_{i}}-\prod_{i=1}^{n}{\left(1-g(t_{T_{i}})\right)}^{w_{i}}}{\prod_{i=1}^{n}{\left(1+g(t_{T_{i}})\right)}^{w_{i}}+\prod_{i=1}^{n}{\left(1-g(t_{T_{i}})\right)}^{w_{i}}}\right),g^{-1}\left(\frac{2\prod_{i=1}^{n}{\left(g(t_{I_{i}})\right)}^{w_{i}}}{\prod_{i=1}^{n}{\left(1+\left(1-g(t_{I_{i}})\right)\right)}^{w_{i}}+\prod_{i=1}^{n}{\left(g(t_{I_{i}})\right)}^{w_{i}}}\right),g^{-1}(\frac{2\prod_{i=1}^{n}{\left(g(t_{F_{i}})\right)}^{w_{i}}}{\prod_{i=1}^{n}{\left(1+\left(1-g(t_{F_{i}})\right)\right)}^{w_{i}}+\prod_{i=1}^{n}{\left(g(t_{F_{i}})\right)}^{w_{i}}})\right); \end{array}$$ (25)

and the *LNHWGM*^*λ*^ operator is degenerated to the linguistic neutrosophic Einstein weighted geometric mean (*LNEWGM*^2^) operator as follows:

$$\begin{array}{l} LNEWGM^{\lambda }(d_{1},d_{2},\cdots ,d_{n})=\\ \left(g^{-1}\left(\frac{2\prod_{i=1}^{n}{\left(g(t_{T_{i}})\right)}^{w_{i}}}{\prod_{i=1}^{n}{\left(1+\left(1-g(t_{T_{i}})\right)\right)}^{w_{i}}+\prod_{i=1}^{n}{\left(g(t_{T_{i}})\right)}^{w_{i}}}\right),g^{-1}\left(\frac{\prod_{i=1}^{n}{\left(1+g(t_{I_{i}})\right)}^{w_{i}}-\prod_{i=1}^{n}{\left(1-g(t_{I_{i}})\right)}^{w_{i}}}{\prod_{i=1}^{n}{\left(1+g(t_{I_{i}})\right)}^{w_{i}}+\prod_{i=1}^{n}{\left(1-g(t_{I_{i}})\right)}^{w_{i}}}\right),g^{-1}\left(\frac{\prod_{i=1}^{n}{\left(1+g(t_{F_{i}})\right)}^{w_{i}}-\prod_{i=1}^{n}{\left(1-g(t_{F_{i}})\right)}^{w_{i}}}{\prod_{i=1}^{n}{\left(1+g(t_{F_{i}})\right)}^{w_{i}}+\prod_{i=1}^{n}{\left(1-g(t_{F_{i}})\right)}^{w_{i}}}\right)\right). \end{array}$$ (26)

**Property 1**. (Commutativity) Given a group of LNNs $d_{i}=(t_{T_{i}},t_{I_{i}},t_{F_{i}})$ (*i* = 1, 2,⋯,*n*), if the permutation of $d_{i}=(t_{T_{i}},t_{I_{i}},t_{F_{i}})$ is $d_{\left(i\right)}=(t_{T\left({}_{i}\right)},t_{I\left({}_{i}\right)},t_{F\left({}_{i}\right)})$, then

$$LNHWAM^{\lambda }(d_{1},d_{2},\cdots ,d_{n})=LNHWAM^{\lambda }(d_{\left(1\right)},d_{\left(2\right)},\cdots ,d_{\left(n\right)}),$$ (27)

$$LNHWGM^{\lambda }(d_{1},d_{2},\cdots ,d_{n})=LNHWGM^{\lambda }(d_{\left(1\right)},d_{\left(2\right)},\cdots ,d_{\left(n\right)}).$$ (28)

**Proof**. Based on Definition 7 and Definition 8, it is clear that the conclusion is true.

**Property 2**. (Idempotency) Suppose $d_{i}=(t_{T_{i}},t_{I_{i}},t_{F_{i}})$(*i* = 1, 2,⋯,*n*) is a collection of LNNs, when *d*_1_ = *d*_2_ = ⋯ = *d*_*n*_ = *d* = (*t*_*T*_, *t*_*I*_, *t*_*F*_), then

$$LNHWAM^{\lambda }(d_{1},d_{2},\cdots ,d_{n})=d,$$ (29)

$$LNHWGM^{\lambda }(d_{1},d_{2},\cdots ,d_{n})=d.$$ (30)

**Proof**.

According to Eq (20),

$$\begin{array}{l} LNHWAM^{\lambda }(d_{1},d_{2},\cdots ,d_{n})=\\ (g^{-1}\left(\frac{\prod_{i=1}^{n}{\left(1+(\lambda -1)(g(t_{T_{i}}))\right)}^{w_{i}}-\prod_{i=1}^{n}{\left(1-g(t_{T_{i}})\right)}^{w_{i}}}{\prod_{i=1}^{n}{\left(1+(\lambda -1)(g(t_{T_{i}}))\right)}^{w_{i}}+(\lambda -1)\prod_{i=1}^{n}{\left(1-g(t_{T_{i}})\right)}^{w_{i}}}\right),g^{-1}(\frac{\lambda \prod_{i=1}^{n}{\left(g(t_{I_{i}})\right)}^{w_{i}}}{\prod_{i=1}^{n}{\left(1+(\lambda -1)\left(1-g(t_{I_{i}})\right)\right)}^{w_{i}}+(\lambda -1)\prod_{i=1}^{n}{\left(g(t_{I_{i}})\right)}^{w_{i}}}),\\ g^{-1}\left(\frac{\lambda \prod_{i=1}^{n}{\left(g(t_{F_{i}})\right)}^{w_{i}}}{\prod_{i=1}^{n}{\left(1+(\lambda -1)\left(1-g(t_{F_{i}})\right)\right)}^{w_{i}}+(\lambda -1)\prod_{i=1}^{n}{\left(g(t_{F_{i}})\right)}^{w_{i}}}\right))\\ =(g^{-1}\left(\frac{\prod_{i=1}^{n}{\left(1+(\lambda -1)(g(t_{T}))\right)}^{w_{i}}-\prod_{i=1}^{n}{\left(1-g(t_{T})\right)}^{w_{i}}}{\prod_{i=1}^{n}{\left(1+(\lambda -1)(g(t_{T}))\right)}^{w_{i}}+(\lambda -1)\prod_{i=1}^{n}{\left(1-g(t_{T})\right)}^{w_{i}}}\right),g^{-1}(\frac{\lambda \prod_{i=1}^{n}{\left(g(t_{I})\right)}^{w_{i}}}{\prod_{i=1}^{n}{\left(1+(\lambda -1)\left(1-g(t_{I})\right)\right)}^{w_{i}}+(\lambda -1)\prod_{i=1}^{n}{\left(g(t_{I})\right)}^{w_{i}}}),\\ \;g^{-1}\left(\frac{\lambda \prod_{i=1}^{n}{\left(g(t_{F})\right)}^{w_{i}}}{\prod_{i=1}^{n}{\left(1+(\lambda -1)\left(1-g(t_{F})\right)\right)}^{w_{i}}+(\lambda -1)\prod_{i=1}^{n}{\left(g(t_{F})\right)}^{w_{i}}}\right))\\ =(g^{-1}\left(\frac{{\left(1+(\lambda -1)(g(t_{T}))\right)}^{\sum_{i=1}^{n}w_{i}}-{\left(1-g(t_{T})\right)}^{\sum_{i=1}^{n}w_{i}}}{{\left(1+(\lambda -1)(g(t_{T}))\right)}^{\sum_{i=1}^{n}w_{i}}+(\lambda -1){\left(1-g(t_{T})\right)}^{\sum_{i=1}^{n}w_{i}}}\right),g^{-1}(\frac{\lambda {\left(g(t_{I})\right)}^{\sum_{i=1}^{n}w_{i}}}{{\left(1+(\lambda -1)\left(1-g(t_{I})\right)\right)}^{\sum_{i=1}^{n}w_{i}}+(\lambda -1){\left(g(t_{I})\right)}^{\sum_{i=1}^{n}w_{i}}}),\\ g^{-1}(\frac{\lambda {\left(g(t_{F})\right)}^{\sum_{i=1}^{n}w_{i}}}{{\left(1+(\lambda -1)\left(1-g(t_{F})\right)\right)}^{\sum_{i=1}^{n}w_{i}}+(\lambda -1){\left(g(t_{F})\right)}^{\sum_{i=1}^{n}w_{i}}}))\\ =\left(g^{-1}\left(\frac{\lambda \cdot (g(t_{T}))}{\lambda }\right),g^{-1}\left(\frac{\lambda \cdot (g(t_{I}))}{\lambda }\right),g^{-1}\left(\frac{\lambda \cdot (g(t_{F}))}{\lambda }\right)\right)\\ =\left(g^{-1}\left(g(t_{T})\right),g^{-1}\left(g(t_{I})\right),g^{-1}\left(g(t_{F})\right)\right)=(t_{T},t_{I},t_{F})=d \end{array}$$ (31)

Similarly, according to Eq (22), it is easy to prove that *LNHWGM*^*λ*^(*d*_1_, *d*_2_,⋯,*d*_*n*_).

Now, this proof is completed.

**Property 3**. (Boundary) Let $d_{i}=(t_{T_{i}},t_{I_{i}},t_{F_{i}})$(*i* = 1, 2,⋯,*n*) be a set of LNNs, if $d^{+}=\left(\underset{i}{\text{max}}(t_{T_{i}}),\underset{i}{\text{min}}(t_{I_{i}}),\underset{i}{\text{min}}(t_{F_{i}})\right)$ and $d^{-}=\left(\underset{i}{\text{min}}(t_{T_{i}}),\underset{i}{\text{max}}(t_{I_{i}}),\underset{i}{\text{max}}(t_{F_{i}})\right)$, then

$$d^{-}\leq LNHWAM^{\lambda }(d_{1},d_{2},\cdots ,d_{n})\leq d^{+},$$ (32)

$$d^{-}\leq LNHWGM^{\lambda }(d_{1},d_{2},\cdots ,d_{n})\leq d^{+}.$$ (33)

**Proof**.

1. As $g(t_{T_{i}})=\frac{t_{T_{i}}}{2s}\in [0,1]$, then
$$\frac{1+(\lambda -1)\left(g\left(\underset{i}{\text{min}}(t_{T_{i}})\right)\right)}{1-g\left(\underset{i}{\text{min}}(t_{T_{i}})\right)}\leq \frac{1+(\lambda -1)(g(t_{T_{i}}))}{1-g(t_{T_{i}})}\leq \frac{1+(\lambda -1)\left(g\left(\underset{i}{\text{max}}(t_{T_{i}})\right)\right)}{1-g\left(\underset{i}{\text{max}}(t_{T_{i}})\right)},$$ (34)
$$\Rightarrow {\left(\frac{1+(\lambda -1)\left(g\left(\underset{i}{\text{min}}(t_{T_{i}})\right)\right)}{1-g\left(\underset{i}{\text{min}}(t_{T_{i}})\right)}\right)}^{w_{i}}\leq {\left(\frac{1+(\lambda -1)(g(t_{T_{i}}))}{1-g(t_{T_{i}})}\right)}^{w_{i}}\leq {\left(\frac{1+(\lambda -1)\left(g\left(\underset{i}{\text{max}}(t_{T_{i}})\right)\right)}{1-g\left(\underset{i}{\text{max}}(t_{T_{i}})\right)}\right)}^{w_{i}},$$ (35)
$$\Rightarrow \prod_{i=1}^{n}{\left(\frac{1+(\lambda -1)\left(g\left(\underset{i}{\text{min}}(t_{T_{i}})\right)\right)}{1-g\left(\underset{i}{\text{min}}(t_{T_{i}})\right)}\right)}^{w_{i}}\leq \prod_{i=1}^{n}{\left(\frac{1+(\lambda -1)(g(t_{T_{i}}))}{1-g(t_{T_{i}})}\right)}^{w_{i}}\leq \prod_{i=1}^{n}{\left(\frac{1+(\lambda -1)\left(g\left(\underset{i}{\text{max}}(t_{T_{i}})\right)\right)}{1-g\left(\underset{i}{\text{max}}(t_{T_{i}})\right)}\right)}^{w_{i}},$$ (36)
$$\Rightarrow \frac{1+(\lambda -1)\left(g\left(\underset{i}{\text{min}}(t_{T_{i}})\right)\right)}{1-g\left(\underset{i}{\text{min}}(t_{T_{i}})\right)}\leq \prod_{i=1}^{n}{\left(\frac{1+(\lambda -1)(g(t_{T_{i}}))}{1-g(t_{T_{i}})}\right)}^{w_{i}}\leq \frac{1+(\lambda -1)\left(g\left(\underset{i}{\text{max}}(t_{T_{i}})\right)\right)}{1-g\left(\underset{i}{\text{max}}(t_{T_{i}})\right)},$$ (37)
$$\Rightarrow \lambda +\frac{\lambda \cdot g\left(\underset{i}{\text{min}}(t_{T_{i}})\right)}{1-g\left(\underset{i}{\text{min}}(t_{T_{i}})\right)}\leq \prod_{i=1}^{n}{\left(\frac{1+(\lambda -1)(g(t_{T_{i}}))}{1-g(t_{T_{i}})}\right)}^{w_{i}}+\lambda -1\leq \lambda +\frac{\lambda \cdot g\left(\underset{i}{\text{max}}(t_{T_{i}})\right)}{1-g\left(\underset{i}{\text{max}}(t_{T_{i}})\right)},$$ (38)
$$\Rightarrow \frac{1}{\lambda +\frac{\lambda \cdot g\left(\underset{i}{\text{max}}(t_{T_{i}})\right)}{1-g\left(\underset{i}{\text{max}}(t_{T_{i}})\right)}}\leq \frac{1}{\prod_{i=1}^{n}{\left(\frac{1+(\lambda -1)(g(t_{T_{i}}))}{1-g(t_{T_{i}})}\right)}^{w_{i}}+\lambda -1}\leq \frac{1}{\lambda +\frac{\lambda \cdot g\left(\underset{i}{\text{min}}(t_{T_{i}})\right)}{1-g\left(\underset{i}{\text{min}}(t_{T_{i}})\right)}},$$ (39)
$$\Rightarrow \frac{1-g\left(\underset{i}{\text{max}}(t_{T_{i}})\right)}{\lambda }\leq \frac{\prod_{i=1}^{n}{\left(1-g(t_{T_{i}})\right)}^{w_{i}}}{\prod_{i=1}^{n}{\left(1+(\lambda -1)\cdot g(t_{T_{i}})\right)}^{w_{i}}+(\lambda -1)\prod_{i=1}^{n}{\left(1-g(t_{T_{i}})\right)}^{w_{i}}}\leq \frac{1-g\left(\underset{i}{\text{min}}(t_{T_{i}})\right)}{\lambda },$$ (40)
$$\Rightarrow 1-g\left(\underset{i}{\text{max}}(t_{T_{i}})\right)\leq \frac{\lambda \prod_{i=1}^{n}{\left(1-g(t_{T_{i}})\right)}^{w_{i}}}{\prod_{i=1}^{n}{\left(1+(\lambda -1)\cdot g(t_{T_{i}})\right)}^{w_{i}}+(\lambda -1)\prod_{i=1}^{n}{\left(1-g(t_{T_{i}})\right)}^{w_{i}}}\leq 1-g\left(\underset{i}{\text{min}}(t_{T_{i}})\right),$$ (41)
$$\Rightarrow g\left(\underset{i}{\text{min}}(t_{T_{i}})\right)\leq \frac{\prod_{i=1}^{n}{\left(1+(\lambda -1)\cdot g(t_{T_{i}})\right)}^{w_{i}}-\prod_{i=1}^{n}{\left(1-g(t_{T_{i}})\right)}^{w_{i}}}{\prod_{i=1}^{n}{\left(1+(\lambda -1)\cdot g(t_{T_{i}})\right)}^{w_{i}}+(\lambda -1)\prod_{i=1}^{n}{\left(1-g(t_{T_{i}})\right)}^{w_{i}}}\leq g\left(\underset{i}{\text{max}}(t_{T_{i}})\right).$$ (42)
2. As $g(t_{I_{i}})=\frac{t_{I_{i}}}{2s}\in [0,1]$, then
$$\frac{1+(\lambda -1)\left(1-g\left(\underset{i}{\text{max}}(t_{I_{i}})\right)\right)}{g\left(\underset{i}{\text{max}}(t_{I_{i}})\right)}\leq \frac{1+(\lambda -1)\left(1-g(t_{I_{i}})\right)}{g(t_{I_{i}})}\leq \frac{1+(\lambda -1)\left(1-g\left(\underset{i}{\text{min}}(t_{I_{i}})\right)\right)}{g\left(\underset{i}{\text{min}}(t_{I_{i}})\right)},$$ (43)
$$\Rightarrow {\left(\frac{1+(\lambda -1)\left(1-g\left(\underset{i}{\text{max}}(t_{I_{i}})\right)\right)}{g\left(\underset{i}{\text{max}}(t_{I_{i}})\right)}\right)}^{w_{i}}\leq {\left(\frac{1+(\lambda -1)\left(1-g(t_{I_{i}})\right)}{g(t_{I_{i}})}\right)}^{w_{i}}\leq {\left(\frac{1+(\lambda -1)\left(1-g\left(\underset{i}{\text{min}}(t_{I_{i}})\right)\right)}{g\left(\underset{i}{\text{min}}(t_{I_{i}})\right)}\right)}^{w_{i}},$$ (44)
$$\Rightarrow \prod_{i=1}^{n}{\left(\frac{1+(\lambda -1)\left(1-g\left(\underset{i}{\text{max}}(t_{I_{i}})\right)\right)}{g\left(\underset{i}{\text{max}}(t_{I_{i}})\right)}\right)}^{w_{i}}\leq \prod_{i=1}^{n}{\left(\frac{1+(\lambda -1)\left(1-g(t_{I_{i}})\right)}{g(t_{I_{i}})}\right)}^{w_{i}}\leq \prod_{i=1}^{n}{\left(\frac{1+(\lambda -1)\left(1-g\left(\underset{i}{\text{min}}(t_{I_{i}})\right)\right)}{g\left(\underset{i}{\text{min}}(t_{I_{i}})\right)}\right)}^{w_{i}},$$ (45)
$$\Rightarrow \frac{1}{g\left(\underset{i}{\text{max}}(t_{I_{i}})\right)}-(\lambda -1)\leq \prod_{i=1}^{n}{\left(\frac{1+(\lambda -1)\left(1-g(t_{I_{i}})\right)}{g(t_{I_{i}})}\right)}^{w_{i}}\leq \frac{1}{g\left(\underset{i}{\text{min}}(t_{I_{i}})\right)}-(\lambda -1),$$ (46)
$$\Rightarrow g\left(\underset{i}{\text{min}}(t_{I_{i}})\right)\leq \frac{1}{\prod_{i=1}^{n}{\left(\frac{1+(\lambda -1)\left(1-g(t_{I_{i}})\right)}{g(t_{I_{i}})}\right)}^{w_{i}}+(\lambda -1)}\leq g\left(\underset{i}{\text{max}}(t_{I_{i}})\right),$$ (47)
$$\Rightarrow g\left(\underset{i}{\text{min}}(t_{I_{i}})\right)\leq \frac{\prod_{i=1}^{n}{\left(g(t_{I_{i}})\right)}^{w_{i}}}{\prod_{i=1}^{n}{\left(1+(\lambda -1)\left(1-g(t_{I_{i}})\right)\right)}^{w_{i}}+(\lambda -1)\prod_{i=1}^{n}{\left(g(t_{I_{i}})\right)}^{w_{i}}}\leq g\left(\underset{i}{\text{max}}(t_{I_{i}})\right).$$ (48)
3. Similarly, as $g(t_{F_{i}})=\frac{t_{F_{i}}}{2s}\in [0,1]$, then
$$\frac{1+(\lambda -1)\left(1-g\left(\underset{i}{\text{max}}(t_{F_{i}})\right)\right)}{g\left(\underset{i}{\text{max}}(t_{F_{i}})\right)}\leq \frac{1+(\lambda -1)\left(1-g(t_{F_{i}})\right)}{g(t_{F_{i}})}\leq \frac{1+(\lambda -1)\left(1-g\left(\underset{i}{\text{min}}(t_{F_{i}})\right)\right)}{g\left(\underset{i}{\text{min}}(t_{F_{i}})\right)},$$ (49)
$$\Rightarrow g\left(\underset{i}{\text{min}}(t_{F_{i}})\right)\leq \frac{\prod_{i=1}^{n}{\left(g(t_{F_{i}})\right)}^{w_{i}}}{\prod_{i=1}^{n}{\left(1+(\lambda -1)\left(1-g(t_{F_{i}})\right)\right)}^{w_{i}}+(\lambda -1)\prod_{i=1}^{n}{\left(g(t_{F_{i}})\right)}^{w_{i}}}\leq g\left(\underset{i}{\text{max}}(t_{F_{i}})\right).$$ (50)

Based on (1)-(3), it is obvious that *d*^−^ ≤ *LNHWAM*^*λ*^ (*d*_1_, *d*_2_,⋯, *d*_*n*_) ≤ *d*^+^. Similarly, it is easy to confirm that *d*^−^ ≤ *LNHWAM*^*λ*^ (*d*_1_, *d*_2_,⋯, *d*_*n*_) ≤ *d*^+^.

Now, this property is proved.

**Property 4**. (Comonotonicity) Assume $d_{i}^{1}=(t_{T_{i}^{1}},t_{I_{i}^{1}},t_{F_{i}^{1}})$ and $d_{i}^{2}=(t_{T_{i}^{2}},t_{I_{i}^{2}},t_{F_{i}^{2}})$(*i* = 1, 2,⋯,*n*) are two arbitrary sets of LNNs, $d_{\left(1\right)}^{1}\leq d_{\left(2\right)}^{1}\leq \cdots \leq d_{\left(n\right)}^{1}\Leftrightarrow \;d_{\left(1\right)}^{2}\leq d_{\left(2\right)}^{2}\leq \cdots \leq d_{\left(n\right)}^{2}$ and $d_{\left(i\right)}^{1}\leq d_{\left(i\right)}^{2}$ for all *i* = 1, 2,⋯,*n*, then

$$LNHWAM^{\lambda }(d_{1}^{1},d_{2}^{1},\cdots ,d_{n}^{1})\leq LNHWAM^{\lambda }(d_{1}^{2},d_{2}^{2},\cdots ,d_{n}^{2}),$$ (51)

$$LNHWGM^{\lambda }(d_{1}^{1},d_{2}^{1},\cdots ,d_{n}^{1})\leq LNHWGM^{\lambda }(d_{1}^{2},d_{2}^{2},\cdots ,d_{n}^{2}).$$ (52)

**Proof**.

Property 1 indicates that $LNHWAM^{\lambda }(d_{1}^{1},d_{2}^{1},\cdots ,d_{n}^{1})=LNHWAM^{\lambda }(d_{\left(1\right)}^{1},d_{\left(2\right)}^{1},\cdots ,d_{\left(n\right)}^{1})$ and $LNHWAM^{\lambda }(d_{1}^{2},d_{2}^{2},\cdots ,d_{n}^{2})=LNHWAM^{\lambda }(d_{\left(1\right)}^{2},d_{\left(2\right)}^{2},\cdots ,d_{\left(n\right)}^{2})$.

Since for all *i* = 1, 2,⋯,*n*, $d_{\left(i\right)}^{1}\leq d_{\left(i\right)}^{2}$ holds. Following Property 3, it can be obtained that $\frac{1+(\lambda -1)(g(t_{T_{\left(i\right)}^{1}}))}{1-g(t_{T_{\left(i\right)}^{1}})}\leq \frac{1+(\lambda -1)(g(t_{T_{\left(i\right)}^{2}}))}{1-g(t_{T_{\left(i\right)}^{2}})}$, $\frac{1+(\lambda -1)(1-g(t_{I_{\left(i\right)}^{2}}))}{g(t_{I_{\left(i\right)}^{2}})}\leq \frac{1+(\lambda -1)(1-g(t_{I_{\left(i\right)}^{1}}))}{g(t_{I_{\left(i\right)}^{1}})}$ and $\frac{1+(\lambda -1)(1-g(t_{F_{\left(i\right)}^{2}}))}{g(t_{F_{\left(i\right)}^{2}})}\leq \frac{1+(\lambda -1)(1-g(t_{F_{\left(i\right)}^{1}}))}{g(t_{F_{\left(i\right)}^{1}})}$. Then, based on Property 3, it can be proved that $LNHWAM^{\lambda }(d_{\left(1\right)}^{1},d_{\left(2\right)}^{1},\cdots ,d_{\left(n\right)}^{1})\leq LNHWAM^{\lambda }(d_{\left(1\right)}^{2},d_{\left(2\right)}^{2},\cdots ,d_{\left(n\right)}^{2})$.

Thus, $LNHWAM^{\lambda }(d_{1}^{1},d_{2}^{1},\cdots ,d_{n}^{1})\leq LNHWAM^{\lambda }(d_{1}^{2},d_{2}^{2},\cdots ,d_{n}^{2})$.

Similarly, it is easy to derive that $LNHWGM^{\lambda }(d_{1}^{1},d_{2}^{1},\cdots ,d_{n}^{1})\leq LNHWGM^{\lambda }(d_{1}^{2},d_{2}^{2},\cdots ,d_{n}^{2})$.

Now, this proof is completed.

### 3.2 Linguistic neutrosophic Hamacher hybrid weighted mean operators

**Definition 9**. If $d_{i}=(t_{T_{i}},t_{I_{i}},t_{F_{i}})$ is a set of LNNs on the linguistic term set *T* = {*t*_*i*_ | *i* ∈ [0,2*s*]}, *d*_(*i*)_ is the *i*-th smallest value of *d*_*i*_, and the weight of the *i*-th ordered position is *w*_*i*_ where *w*_*i*_ ∈ [0, 1](*i* = 1, 2,⋯,*n*) and *w*_1_ + *w*_2_ +⋯+*w*_*n*_ = 1. Then the linguistic neutrosophic Hamacher ordered weighted arithmetic mean (*LNHOWAM*^*λ*^) operator is

$$LNHOWAM^{\lambda }(d_{1},d_{2},\cdots ,d_{n})={⊕}_{i=1}^{n}(w_{i}d_{\left(i\right)})=w_{1}d_{\left(1\right)}⊕w_{2}d_{\left(2\right)}⊕\cdots ⊕w_{n}d_{\left(n\right)}.$$ (53)

**Definition 10**. Assume $d_{i}=(t_{T_{i}},t_{I_{i}},t_{F_{i}})$ is a group of LNNs on the linguistic term set *T* = {*t*_*i*_ | *i* ∈ [0,2*s*]}, *d*_(*i*)_ is the *i*-th smallest value of *d*_*i*_, and the weight of the *i*-th ordered position is *w*_*i*_ where *w*_*i*_ ∈ [0, 1] (*i* = 1, 2,⋯,*n*) and *w*_1_ + *w*_2_ +⋯+*w*_*n*_ = 1. Then the linguistic neutrosophic Hamacher ordered weighted geometric mean (*LNHOWGM*^*λ*^) operator is

$$LNHOWGM^{\lambda }(d_{1},d_{2},\cdots ,d_{n})={⊗}_{i=1}^{n}{\left(d_{\left(i\right)}\right)}^{w_{i}}={\left(d_{\left(1\right)}\right)}^{w_{1}}⊗{\left(d_{\left(2\right)}\right)}^{w_{2}}⊗\cdots ⊗{\left(d_{\left(n\right)}\right)}^{w_{n}}.$$ (54)

If the weights of LNNs and ordered positions are taken into account at the same time, several hybrid weighted aggregation operators can be obtained as follows.

**Definition 11**. Let $d_{i}=(t_{T_{i}},t_{I_{i}},t_{F_{i}})$ be a set of LNNs on the linguistic term set *T* = {*t*_*i*_ | *i* ∈ [0,2*s*]}, *ω*_(*i*)_*d*_(*i*)_ is the *i*-th smallest value of *ω*_*i*_*d*_*i*_, and two weight vectors are *ω* = (*ω*_1_, *ω*_2_,⋯ *ω*_*n*_) and *w* = (*w*_1_, *w*_2_,⋯, *w*_*n*_) where *ω*_*i*_, *w*_*i*_ ∈ [0, 1] (*i* = 1, 2,⋯,*n*) and $\sum_{i=1}^{n}\omega_{i}=\sum_{i=1}^{n}w_{i}=1$. Then the linguistic neutrosophic Hamacher hybrid weighted arithmetic mean (*LNHHWAM*^*λ*^) operator is

$$LNHHWAM^{\lambda }(d_{1},d_{2},\cdots ,d_{n})={⊕}_{i=1}^{n}\left(\frac{\omega_{\left(i\right)}w_{i}}{\sum_{i=1}^{n}\omega_{\left(i\right)}w_{i}}d_{\left(i\right)}\right)=\frac{\omega_{\left(1\right)}w_{1}}{\sum_{i=1}^{n}\omega_{\left(i\right)}w_{i}}d_{\left(1\right)}⊕\frac{\omega_{\left(2\right)}w_{2}}{\sum_{i=1}^{n}\omega_{\left(i\right)}w_{i}}d_{\left(2\right)}⊕\cdots ⊕\frac{\omega_{\left(n\right)}w_{n}}{\sum_{i=1}^{n}\omega_{\left(i\right)}w_{i}}d_{\left(n\right)}.$$ (55)

**Theorem 3**. If *T* = {*t*_*i*_ | *i* ∈ [0,2*s*]} is a continuous linguistic term set, and $d_{i}=(t_{T_{i}},t_{I_{i}},t_{F_{i}})$ is a set of LNNs, then the aggregated value by using the *LNHHWAM*^*λ*^ operator is still a LNN, which is shown as follows:

$$\begin{array}{l} LNHHWAM^{\lambda }(d_{1},d_{2},\cdots ,d_{n})=\\ (g^{-1}\left(\frac{\prod_{i=1}^{n}{\left(1+(\lambda -1)\cdot g(t_{T_{\left(i\right)}})\right)}^{\frac{\omega_{\left(i\right)}w_{i}}{\sum_{i=1}^{n}\omega_{\left(i\right)}w_{i}}}-\prod_{i=1}^{n}{\left(1-g(t_{T_{\left(i\right)}})\right)}^{\frac{\omega_{\left(i\right)}w_{i}}{\sum_{i=1}^{n}\omega_{\left(i\right)}w_{i}}}}{\prod_{i=1}^{n}{\left(1+(\lambda -1)\cdot g(t_{T_{\left(i\right)}})\right)}^{\frac{\omega_{\left(i\right)}w_{i}}{\sum_{i=1}^{n}\omega_{\left(i\right)}w_{i}}}+(\lambda -1)\prod_{i=1}^{n}{\left(1-g(t_{T_{\left(i\right)}})\right)}^{\frac{\omega_{\left(i\right)}w_{i}}{\sum_{i=1}^{n}\omega_{\left(i\right)}w_{i}}}}\right),g^{-1}\left(\frac{\lambda \prod_{i=1}^{n}{\left(g(t_{I_{\left(i\right)}})\right)}^{\frac{\omega_{\left(i\right)}w_{i}}{\sum_{i=1}^{n}\omega_{\left(i\right)}w_{i}}}}{\prod_{i=1}^{n}{\left(1+(\lambda -1)\left(1-g(t_{I_{\left(i\right)}})\right)\right)}^{\frac{\omega_{\left(i\right)}w_{i}}{\sum_{i=1}^{n}\omega_{\left(i\right)}w_{i}}}+(\lambda -1)\prod_{i=1}^{n}{\left(g(t_{I_{\left(i\right)}})\right)}^{\frac{\omega_{\left(i\right)}w_{i}}{\sum_{i=1}^{n}\omega_{\left(i\right)}w_{i}}}}\right),\\ g^{-1}\left(\frac{\lambda \prod_{i=1}^{n}{\left(g(t_{F_{\left(i\right)}})\right)}^{\frac{\omega_{\left(i\right)}w_{i}}{\sum_{i=1}^{n}\omega_{\left(i\right)}w_{i}}}}{\prod_{i=1}^{n}{\left(1+(\lambda -1)\left(1-g(t_{F_{\left(i\right)}})\right)\right)}^{\frac{\omega_{\left(i\right)}w_{i}}{\sum_{i=1}^{n}\omega_{\left(i\right)}w_{i}}}+(\lambda -1)\prod_{i=1}^{n}{\left(g(t_{F_{\left(i\right)}})\right)}^{\frac{\omega_{\left(i\right)}w_{i}}{\sum_{i=1}^{n}\omega_{\left(i\right)}w_{i}}}}\right)) \end{array}.$$ (56)

**Definition 12**. Suppose $d_{i}=(t_{T_{i}},t_{I_{i}},t_{F_{i}})$ is a set of LNNs on the linguistic term set *T* = {*t*_*i*_ | *i* ∈ [0,2*s*]}, ${\left(d_{\left(i\right)}\right)}^{\omega_{\left(i\right)}}$ is the *i*-th smallest value of ${\left(d_{i}\right)}^{\omega_{i}}$, and two weight vectors are *w* = (*w*_1_, *w*_2_,⋯, *w*_*n*_) and *w* = (*w*_1_, *w*_2_,⋯, *w*_*n*_) where *ω*_*i*_, *w*_*i*_ ∈ [0, 1] (*i* = 1, 2,⋯,*n*) and $\sum_{i=1}^{n}\omega_{i}=\sum_{i=1}^{n}w_{i}=1$. Then the linguistic neutrosophic Hamacher hybrid weighted geometric mean (*LNHHWGM*^*λ*^) operator is

$$LNHHWGM^{\lambda }(d_{1},d_{2},\cdots ,d_{n})={⊗}_{i=1}^{n}{\left(d_{\left(i\right)}\right)}^{\frac{\omega_{\left(i\right)}w_{i}}{\sum_{i=1}^{n}\omega_{\left(i\right)}w_{i}}}={\left(d_{\left(1\right)}\right)}^{\frac{\omega_{\left(1\right)}w_{1}}{\sum_{i=1}^{n}\omega_{\left(i\right)}w_{i}}}⊗{\left(d_{\left(2\right)}\right)}^{\frac{\omega_{\left(2\right)}w_{2}}{\sum_{i=1}^{n}\omega_{\left(i\right)}w_{i}}}⊗\cdots ⊗{\left(d_{\left(n\right)}\right)}^{\frac{\omega_{\left(n\right)}w_{n}}{\sum_{i=1}^{n}\omega_{\left(i\right)}w_{i}}}.$$ (57)

**Theorem 4**. If *T* = {*t*_*i*_ | *i* ∈ [0,2*s*]} is a continuous linguistic term set, and $d_{i}=(t_{T_{i}},t_{I_{i}},t_{F_{i}})$ is a set of LNNs, then the aggregated value by using the *LNHHWGM*^*λ*^ operator is still a LNN, which is shown as follows:

$$\begin{array}{l} LNHHWGM^{\lambda }(d_{1},d_{2},\cdots ,d_{n})=\\ (g^{-1}\left(\frac{\lambda \prod_{i=1}^{n}{\left(g(t_{T_{\left(i\right)}})\right)}^{\frac{\omega_{\left(i\right)}w_{i}}{\sum_{i=1}^{n}\omega_{\left(i\right)}w_{i}}}}{\prod_{i=1}^{n}{\left(1+(\lambda -1)\left(1-g(t_{T_{\left(i\right)}})\right)\right)}^{\frac{\omega_{\left(i\right)}w_{i}}{\sum_{i=1}^{n}\omega_{\left(i\right)}w_{i}}}+(\lambda -1)\prod_{i=1}^{n}{\left(g(t_{T_{\left(i\right)}})\right)}^{\frac{\omega_{\left(i\right)}w_{i}}{\sum_{i=1}^{n}\omega_{\left(i\right)}w_{i}}}}\right),g^{-1}\left(\frac{\prod_{i=1}^{n}{\left(1+(\lambda -1)\cdot g(t_{I_{\left(i\right)}})\right)}^{\frac{\omega_{\left(i\right)}w_{i}}{\sum_{i=1}^{n}\omega_{\left(i\right)}w_{i}}}-\prod_{i=1}^{n}{\left(1-g(t_{I_{\left(i\right)}})\right)}^{\frac{\omega_{\left(i\right)}w_{i}}{\sum_{i=1}^{n}\omega_{\left(i\right)}w_{i}}}}{\prod_{i=1}^{n}{\left(1+(\lambda -1)\cdot g(t_{I_{\left(i\right)}})\right)}^{\frac{\omega_{\left(i\right)}w_{i}}{\sum_{i=1}^{n}\omega_{\left(i\right)}w_{i}}}+(\lambda -1)\prod_{i=1}^{n}{\left(1-g(t_{I_{\left(i\right)}})\right)}^{\frac{\omega_{\left(i\right)}w_{i}}{\sum_{i=1}^{n}\omega_{\left(i\right)}w_{i}}}}\right),\\ g^{-1}\left(\frac{\prod_{i=1}^{n}{\left(1+(\lambda -1)\cdot g(t_{F_{\left(i\right)}})\right)}^{\frac{\omega_{\left(i\right)}w_{i}}{\sum_{i=1}^{n}\omega_{\left(i\right)}w_{i}}}-\prod_{i=1}^{n}{\left(1-g(t_{F_{\left(i\right)}})\right)}^{\frac{\omega_{\left(i\right)}w_{i}}{\sum_{i=1}^{n}\omega_{\left(i\right)}w_{i}}}}{\prod_{i=1}^{n}{\left(1+(\lambda -1)\cdot g(t_{F_{\left(i\right)}})\right)}^{\frac{\omega_{\left(i\right)}w_{i}}{\sum_{i=1}^{n}\omega_{\left(i\right)}w_{i}}}+(\lambda -1)\prod_{i=1}^{n}{\left(1-g(t_{F_{\left(i\right)}})\right)}^{\frac{\omega_{\left(i\right)}w_{i}}{\sum_{i=1}^{n}\omega_{\left(i\right)}w_{i}}}}\right)) \end{array}.$$ (58)

Note that when *λ* = 1, the *LNHHWAM*^*λ*^ operator is degenerated to the linguistic neutrosophic Algebraic hybrid weighted arithmetic mean (*LNAHWAM*^1^) operator as follows:

$$LNAHWAM^{1}(d_{1},d_{2},\cdots ,d_{n})=\left(g^{-1}\left(1-\prod_{i=1}^{n}{\left(1-g(t_{T_{i}})\right)}^{\frac{\omega_{\left(i\right)}w_{i}}{\sum_{i=1}^{n}\omega_{\left(i\right)}w_{i}}}\right),g^{-1}\left(\prod_{i=1}^{n}{\left(g(t_{I_{i}})\right)}^{\frac{\omega_{\left(i\right)}w_{i}}{\sum_{i=1}^{n}\omega_{\left(i\right)}w_{i}}}\right),g^{-1}\left({\prod_{i=1}^{n}\left(g(t_{F_{i}})\right)}^{\frac{\omega_{\left(i\right)}w_{i}}{\sum_{i=1}^{n}\omega_{\left(i\right)}w_{i}}}\right)\right);$$ (59)

and the *LNHHWGM*^*λ*^ operator is degenerated to the linguistic neutrosophic Algebraic hybrid weighted geometric mean (*LNAHWGM*^1^) operator as follows:

$$LNAHWGM^{1}(d_{1},d_{2},\cdots ,d_{n})=\left(g^{-1}\left(\prod_{i=1}^{n}{\left(g(t_{T_{i}})\right)}^{\frac{\omega_{\left(i\right)}w_{i}}{\sum_{i=1}^{n}\omega_{\left(i\right)}w_{i}}}\right),g^{-1}\left(1-\prod_{i=1}^{n}{\left(1-g(t_{I_{i}})\right)}^{\frac{\omega_{\left(i\right)}w_{i}}{\sum_{i=1}^{n}\omega_{\left(i\right)}w_{i}}}\right),g^{-1}\left(1-\prod_{i=1}^{n}{\left(1-g(t_{F_{i}})\right)}^{\frac{\omega_{\left(i\right)}w_{i}}{\sum_{i=1}^{n}\omega_{\left(i\right)}w_{i}}}\right)\right).$$ (60)

When *λ* = 2, the *LNHHWAM*^*λ*^ operator is degenerated to the linguistic neutrosophic Einstein hybrid weighted arithmetic mean (*LNEHWAM*^2^) operator as follows:

$$\begin{array}{l} LNEHWAM^{2}(d_{1},d_{2},\cdots ,d_{n})=\\ (g^{-1}\left(\frac{\prod_{i=1}^{n}{\left(1+g(t_{T_{i}})\right)}^{\frac{\omega_{\left(i\right)}w_{i}}{\sum_{i=1}^{n}\omega_{\left(i\right)}w_{i}}}-\prod_{i=1}^{n}{\left(1-g(t_{T_{i}})\right)}^{\frac{\omega_{\left(i\right)}w_{i}}{\sum_{i=1}^{n}\omega_{\left(i\right)}w_{i}}}}{\prod_{i=1}^{n}{\left(1+g(t_{T_{i}})\right)}^{\frac{\omega_{\left(i\right)}w_{i}}{\sum_{i=1}^{n}\omega_{\left(i\right)}w_{i}}}+\prod_{i=1}^{n}{\left(1-g(t_{T_{i}})\right)}^{\frac{\omega_{\left(i\right)}w_{i}}{\sum_{i=1}^{n}\omega_{\left(i\right)}w_{i}}}}\right),g^{-1}\left(\frac{2\prod_{i=1}^{n}{\left(g(t_{I_{i}})\right)}^{\frac{\omega_{\left(i\right)}w_{i}}{\sum_{i=1}^{n}\omega_{\left(i\right)}w_{i}}}}{\prod_{i=1}^{n}{\left(1+\left(1-g(t_{I_{i}})\right)\right)}^{\frac{\omega_{\left(i\right)}w_{i}}{\sum_{i=1}^{n}\omega_{\left(i\right)}w_{i}}}+\prod_{i=1}^{n}{\left(g(t_{I_{i}})\right)}^{\frac{\omega_{\left(i\right)}w_{i}}{\sum_{i=1}^{n}\omega_{\left(i\right)}w_{i}}}}\right),\\ g^{-1}\left(\frac{2\prod_{i=1}^{n}{\left(g(t_{F_{i}})\right)}^{\frac{\omega_{\left(i\right)}w_{i}}{\sum_{i=1}^{n}\omega_{\left(i\right)}w_{i}}}}{\prod_{i=1}^{n}{\left(1+\left(1-g(t_{F_{i}})\right)\right)}^{\frac{\omega_{\left(i\right)}w_{i}}{\sum_{i=1}^{n}\omega_{\left(i\right)}w_{i}}}+\prod_{i=1}^{n}{\left(g(t_{F_{i}})\right)}^{\frac{\omega_{\left(i\right)}w_{i}}{\sum_{i=1}^{n}\omega_{\left(i\right)}w_{i}}}}\right)) \end{array};$$ (61)

and the *LNHHWGM*^*λ*^ operator is degenerated to the linguistic neutrosophic Einstein hybrid weighted geometric mean (*LNEHWGM*^*λ*^) operator as follows:

$$\begin{array}{l} LNEHWGM^{\lambda }(d_{1},d_{2},\cdots ,d_{n})=\\ (g^{-1}\left(\frac{2\prod_{i=1}^{n}{\left(g(t_{T_{i}})\right)}^{\frac{\omega_{\left(i\right)}w_{i}}{\sum_{i=1}^{n}\omega_{\left(i\right)}w_{i}}}}{\prod_{i=1}^{n}{\left(1+\left(1-g(t_{T_{i}})\right)\right)}^{\frac{\omega_{\left(i\right)}w_{i}}{\sum_{i=1}^{n}\omega_{\left(i\right)}w_{i}}}+\prod_{i=1}^{n}{\left(g(t_{T_{i}})\right)}^{\frac{\omega_{\left(i\right)}w_{i}}{\sum_{i=1}^{n}\omega_{\left(i\right)}w_{i}}}}\right),g^{-1}\left(\frac{\prod_{i=1}^{n}{\left(1+g(t_{I_{i}})\right)}^{\frac{\omega_{\left(i\right)}w_{i}}{\sum_{i=1}^{n}\omega_{\left(i\right)}w_{i}}}-\prod_{i=1}^{n}{\left(1-g(t_{I_{i}})\right)}^{\frac{\omega_{\left(i\right)}w_{i}}{\sum_{i=1}^{n}\omega_{\left(i\right)}w_{i}}}}{\prod_{i=1}^{n}{\left(1+g(t_{I_{i}})\right)}^{\frac{\omega_{\left(i\right)}w_{i}}{\sum_{i=1}^{n}\omega_{\left(i\right)}w_{i}}}+\prod_{i=1}^{n}{\left(1-g(t_{I_{i}})\right)}^{\frac{\omega_{\left(i\right)}w_{i}}{\sum_{i=1}^{n}\omega_{\left(i\right)}w_{i}}}}\right),\\ g^{-1}\left(\frac{\prod_{i=1}^{n}{\left(1+g(t_{F_{i}})\right)}^{\frac{\omega_{\left(i\right)}w_{i}}{\sum_{i=1}^{n}\omega_{\left(i\right)}w_{i}}}-\prod_{i=1}^{n}{\left(1-g(t_{F_{i}})\right)}^{\frac{\omega_{\left(i\right)}w_{i}}{\sum_{i=1}^{n}\omega_{\left(i\right)}w_{i}}}}{\prod_{i=1}^{n}{\left(1+g(t_{F_{i}})\right)}^{\frac{\omega_{\left(i\right)}w_{i}}{\sum_{i=1}^{n}\omega_{\left(i\right)}w_{i}}}+\prod_{i=1}^{n}{\left(1-g(t_{F_{i}})\right)}^{\frac{\omega_{\left(i\right)}w_{i}}{\sum_{i=1}^{n}\omega_{\left(i\right)}w_{i}}}}\right)) \end{array}.$$ (62)

It is clear that the *LNHOWAM*^*λ*^, *LNHOWGM*^*λ*^, *LNHOWAM*^*λ*^, *LNHOWAM*^*λ*^, *LNHOWAM*^*λ*^ and *LNHOWAM*^*λ*^ operators also satisfy the commutativity, idempotency, boundary and commonotonicity properties. Since the proofs of these properties are similar to that for the *LNHWAM*^*λ*^ operators and *LNHWGM*^*λ*^ operators, in order to save space, they are left out in this paper.

## 4. A method for decision making with linguistic neutrosophic information

In this section, a method for decision making with linguistic neutrosophic information is suggested to handle linguistic neutrosophic decision making issues.

### 4.1 Problem description

A multi-criteria linguistic neutrosophic decision making problem can be described as follows:

*α* = {*α*_1_, *α*_2_,⋯, *α*_*m*_}, which is indexed by *x* and *m* ≥ 2, is a collection of alternatives for experts.
*β* = {*β*_1_, *β*_2_,⋯, *β*_*n*_}, which is indexed by *y* and *n* ≥ 2, is a group of criteria for each alternative.
*ω* = (*ω*_1_, *ω*_2_,⋯ *ω*_*n*_) and *w* = (*w*_1_, *w*_2_,⋯, *w*_*n*_) are respectively the criteria and ordered position weight vectors, and both of them are completely unknown for decision makers.
*D* = (*d*_*xy*_)_*m*×*n*_, which is provided by some decision makers, is an original evaluation matrix.
$d_{xy}=(t_{Td_{xy}},t_{Id_{xy}},t_{Fd_{xy}})$, which is in the form of LNNs, is the assessment value of alternative *α*_*x*_ relating to criteria *β*_*y*_; $t_{Td_{xy}}$, $t_{Id_{xy}}$ and $t_{Fd_{xy}}$, which are acquired from a certain linguistic term set *T* = {*t*_*i*_ | *i* ∈ [0,2*s*]} (*s* > 0), are three independent linguistic terms.

### 4.2 Decision making procedures

In order to pick out the ideal alternative, a linguistic neutrosophic decision making method based on Hamacher aggregation operators is presented. The decision making procedures are shown as follows.

**Step 1:** Normalize the original decision making matrix.
  For a certain evaluation matrix, when there are two types of criteria, namely, the benefit and cost criteria, they need to be changed into the same type for convenience. The transformation equation is
$$e_{xy}=\{\begin{array}{cc} \left(t_{Td_{xy}},t_{Id_{xy}},t_{Fd_{xy}}\right) & for\;benefit\;criteria\;\beta_{y}\\ \left(t_{2s-Td_{xy}},t_{2s-Id_{xy}},t_{2s-Fd_{xy}}\right) & for\;\text{cos}t\;criteria\;\beta_{y} \end{array}.$$ (63)
  Then, the standardized linguistic neutrosophic decision making matrix is $E={\left(e_{xy}\right)}_{m\times n}={\left(t_{Te_{xy}},t_{Ie_{xy}},t_{Fe_{xy}}\right)}_{m\times n}$.
**Step 2:** Determine the weights of criteria.
  The idea of criteria weight determination model is that when the deviation of evaluation values among all alternatives under a certain criteria is big, it means such criterion can greatly affect the ranking order. Thus, a large weight value *ω*_*y*_ can be assigned to this criterion. The calculation equations can be expressed as
$$K_{y}=\sum_{x=1}^{m-1}\sum_{z=x+1}^{m}\left(|\frac{4s+Te_{xy}-Ie_{xy}-Fe_{xy}}{6s}-\frac{4s+Te_{zy}-Ie_{zy}-Fe_{zy}}{6s}|\right),$$ (64)
$$L_{y}=\sum_{x=1}^{m-1}\sum_{z=x+1}^{m}\left(|\frac{Te_{xy}-Fe_{xy}}{2s}-\frac{Te_{zy}-Fe_{zy}}{2s}|\right),$$ (65)
$$\omega_{y}=\frac{K_{y}+L_{y}}{\sum_{y=1}^{n}\left(K_{y}+L_{y}\right)},$$ (66)
  where *K*_*y*_ means the deviation of score function values among all alternatives under criterion *β*_*y*_, *L*_*y*_ means the deviation of accuracy function values among all alternatives under criterion *β*_*y*_, and *W*_*y*_ is the normalized weight value of criterion *β*_*y*_ based on Eqs (64) and (65).
**Step 3:** Calculate the weights of ordered positions.
  Suppose $G={\left(g_{xy}\right)}_{m\times n}={\left(t_{Tg_{xy}},t_{Ig_{xy}},t_{Fg_{xy}}\right)}_{m\times n}$ is a weighted linguistic neutrosophic decision making matrix of *E*, where *g*_*xy*_ = *w*_*y*_*e*_*xy*_ or $g_{xy}={\left(e_{xy}\right)}^{\omega_{y}}$, and *g*_*xy*_ is ranked in an increasing order as *g*_*x*(1)_ ≤*g*_*x*(2)_ ≤⋯ ≤*g*_*x*(*n*)_, then the weights of the ordered positions can be calculated with
$$w_{y}=\frac{1/\sum_{x=1}^{m}h_{x(y)}}{\sum_{y=1}^{n}\left(1/\sum_{x=1}^{m}h_{x(y)}\right)},$$ (67)
  where $h_{x(y)}=\{\begin{array}{c} \frac{4s-Tg_{x(y)}+Ig_{x(y)}+Fg_{x(y)}}{6s}\;y\leq \frac{n+1}{2}\\ \frac{4s+Tg_{x(y)}-Ig_{x(y)}-Fg_{x(y)}}{6s}\;y>\frac{n+1}{2} \end{array}$(*y* = 1, 2, ⋯,*n*).
**Step 4:** Compute the comprehensive preference values.
  The *LNHHWAM*^*λ*^ operator or *LNHHWGM*^*λ*^ operator is suggested to compute the comprehensive preference value *e*_*x*_(*x* = 1, 2,⋯,*m*) of each alternative in matrix *E*.
**Step 5:** Obtain the optimal alternative.
  The ranking result can be obtained by calculating the score function and accuracy function values of *e*_*x*_.

## 5. Case study

In this section, a case is studied by adopting the presented method based on linguistic neutrosophic Hamacher aggregation operators to assess land reclamation schemes in mine area.

### 5.1 Project profile

The Kaiyang phosphate mine, which is located in Jinzhong Town, Guiyang City, Guizhou Province, China, is composed of Maluping, Qincaichong, Yongshaba and Shabatu sections. It has a history of nearly 60 years, and the proven phosphate reserve is 1.08 billion tons. With the increase of demand for phosphate rock, the mining intensity is rising greatly, and the annual output is up to 8 million tons. However, considerable land resources are destroyed due to the large-scale mining. Until the end of 2010, the cultivated area destroyed only by collapse of mountain exceeded 15 hm^2^ [47]. Besides, a huge amount of waste residue is produced, which not only occupies large tracts of land, but also destroys ecological environment and induce geological disasters. Therefore, it is very important and urgent to select an optimal land reclamation scheme for Kaiyang phosphate mine to achieve sustainable development.

### 5.2 Evaluation criteria system

Determining the evaluation criteria is necessary and vital for the evaluation of land reclamation schemes. According to the specific characteristics of mine areas, four criteria are selected, including the technology, economy, environment and efficiency. More details are shown in Table 1.

**Table 1:** Evaluation criteria of land reclamation schemes.

Primary indicators	Benefit/Cost	Descriptions
Technology *β*_1_	Benefit	It indicates the technological feasibility of schemes, which includes local climate, terrain and soil conditions.
Economy *β*_2_	Cost	It indicates the predicted economic cost, which includes materials, labor, equipment and maintenance fees.
Environment *β*_3_	Benefit	It indicates the comprehensive environment effect of schemes, which includes land fertility, species diversity and ecological environment quality.
Efficiency *β*_4_	Benefit	It indicates the expected efficiency of schemes, which includes labor intensity and project duration.

### 5.3 Selecting optimal land reclamation scheme

After preliminary investigation, managers in a mine intend to make appraisals among four land reclamation schemes and then select the optimal alternative. The linguistic term set *T* = {*t*_*i*_ | *i* ∈[0, 6]} is utilized, where *t*_0_ = *very low*, *t*_1_ = *low*, *t*_2_ = *a little low*, *t*_3_ = *medium*, *t*_4_ = *a little high*, *t*_5_ = *high* and *t*_6_ = *very high*. Then, the original evaluation matrix provided by experts is shown in Table 2.

**Table 2:** Original decision making matrix *D*.

*D*	*β*_1_	*β*_2_	*β*_3_	*β*_4_
*α*_1_	(*t*_4_, *t*_2_, *t*_0_)	(*t*_1_, *t*_3_, *t*_5_)	(*t*_3_, *t*_2_, *t*_3_)	(*t*_6_, *t*_1_, *t*_2_)
*α*_2_	(*t*_3_, *t*_3_, *t*_2_)	(*t*_2_, *t*_4_, *t*_3_)	(*t*_4_, *t*_1_, *t*_1_)	(*t*_5_, *t*_0_, *t*_2_)
*α*_3_	(*t*_5_, *t*_1_, *t*_1_)	(*t*_3_, *t*_5_, *t*_4_)	(*t*_6_, *t*_3_, *t*_3_)	(*t*_3_, *t*_2_, *t*_1_)
*α*_4_	(*t*_6_, *t*_3_, *t*_2_)	(*t*_1_, *t*_4_, *t*_3_)	(*t*_5_, *t*_1_, *t*_0_)	(*t*_4_, *t*_3_, *t*_2_)

Subsequently, the linguistic neutrosophic decision making method suggested in Section 4 is applied to obtain the ranking result of these schemes.

**Step 1:** Normalize the original decision making matrix.
  Since *β*_2_ belongs to the cost criterion whereas other three indicators are benefit criteria, *β*_2_ is transferred by Eq (63). Then, the normalized decision making matrix is attained (See Table 3).
**Step 2:** Determine the weights of criteria.
  Based on Eqs (64)–(66), the weights of criteria are calculated, and *K*_1_ ≈ 0.8889, *K*_2_ ≈ 0.3333, *K*_3_ ≈ 1.1111, *K*_4_ ≈ 0.8333, *L*_1_ ≈ 1.5000, *L*_2_ ≈ 1.6667, *L*_3_ ≈ 2.5000, *L*_4_ ≈ 1.1667, *w*_1_ ≈ 0.24, *w*_2_ ≈ 0.20, *w*_3_ ≈ 0.36 and *w*_4_ ≈ 0.20.
**Step 3:** Calculate the weights of ordered positions.
  Assume *g*_*xy*_ = *w*_*y*_*e*_*xy*_ with *λ* = 0.1 is used to obtain the weighted linguistic neutrosophic decision making matrix (See Table 4), then according to Eq (67), the weights of ordered positions are calculated as: *w*_1_ ≈ 0.17, *w*_2_ ≈ 0.18, *w*_3_ ≈ 0.35 and *w*_4_ ≈ 0.30.
**Step 4:** Compute the comprehensive preference values.
  According to Eq (56), the *LNHHWAM*^*λ*^ operator with *λ* = 1 is utilized to aggregate each row of the normalized matrix *E*. Thereafter, the comprehensive preference values of alternatives are calculated as: *e*_1_ = (*t*_6_, *t*_1.79_, *t*_0_), *e*_2_ = (*t*_4.18_, *t*_0_, *t*_1.52_), *e*_3_ = (*t*_6_, *t*_1.73_, *t*_1.72_) and *e*_4_ = (*t*_6_, *t*_1.80_, *t*_0_).
**Step 5:** Obtain the optimal alternative.
  Based on Definition 4, the score function values are obtained as: *U*(*e*_1_) ≈ 0.9007, *U*(*e*_2_) ≈ 0.8147, *U*(*e*_3_) ≈ 0.8083 and *U*(*e*_4_) ≈ 0.8998. Since *U*(*e*_1_) > *U*(*e*_4_) > *U*(*e*_2_) > *U*(*e*_3_), then *α*_1_ ≻ *α*_4_ ≻ *α*_2_ ≻ *α*_3_ and the best alternative is *α*_1_.

**Table 3:** Normalized decision making matrix *E*.

*E*	*β*_1_	*β*_2_	*β*_3_	*β*_4_
*α*_1_	(*t*_4_, *t*_2_, *t*_0_)	(*t*_5_, *t*_3_, *t*_1_)	(*t*_3_, *t*_2_, *t*_3_)	(*t*_6_, *t*_1_, *t*_2_)
*α*_2_	(*t*_3_, *t*_3_, *t*_2_)	(*t*_4_, *t*_2_, *t*_3_)	(*t*_4_, *t*_1_, *t*_1_)	(*t*_5_, *t*_0_, *t*_2_)
*α*_3_	(*t*_5_, *t*_1_, *t*_1_)	(*t*_3_, *t*_1_, *t*_2_)	(*t*_6_, *t*_3_, *t*_3_)	(*t*_3_, *t*_2_, *t*_1_)
*α*_4_	(*t*_6_, *t*_3_, *t*_2_)	(*t*_5_, *t*_2_, *t*_3_)	(*t*_5_, *t*_1_, *t*_0_)	(*t*_4_, *t*_3_, *t*_2_)

**Table 4:** Weighted decision making matrix *G*.

*G*	*β*_1_	*β*_2_	*Β*_3_	*β*_4_
*α*_1_	(*t*_1.85_, *t*_4.15_, *t*_0_)	(*t*_2.85_, *t*_5.03_, *t*_3.25_)	(*t*_1.55_, *t*_3.57_, *t*_4.45_)	(*t*_6_, *t*_3.25_, *t*_4.38_)
*α*_2_	(*t*_1.13_, *t*_4.87_, *t*_4.15_)	(*t*_1.62_, *t*_4.38_, *t*_5.03_)	(*t*_2.43_, *t*_2.33_, *t*_2.33_)	(*t*_2.75_, *t*_0_, *t*_4.38_)
*α*_3_	(*t*_3.03_, *t*_2.97_, *t*_2.97_)	(*t*_0.97_, *t*_3.25_, *t*_4.38_)	(*t*_6_, *t*_4.45_, *t*_4.45_)	(*t*_0.97_, *t*_4.38_, *t*_3.25_)
*α*_4_	(*t*_6_, *t*_4.87_, *t*_4.15_)	(*t*_2.75_, *t*_4.38_, *t*_5.03_)	(*t*_3.67_, *t*_2.33_, *t*_0_)	(*t*_1.62_, *t*_5.03_, *t*_4.38_)

**Fig 1:**
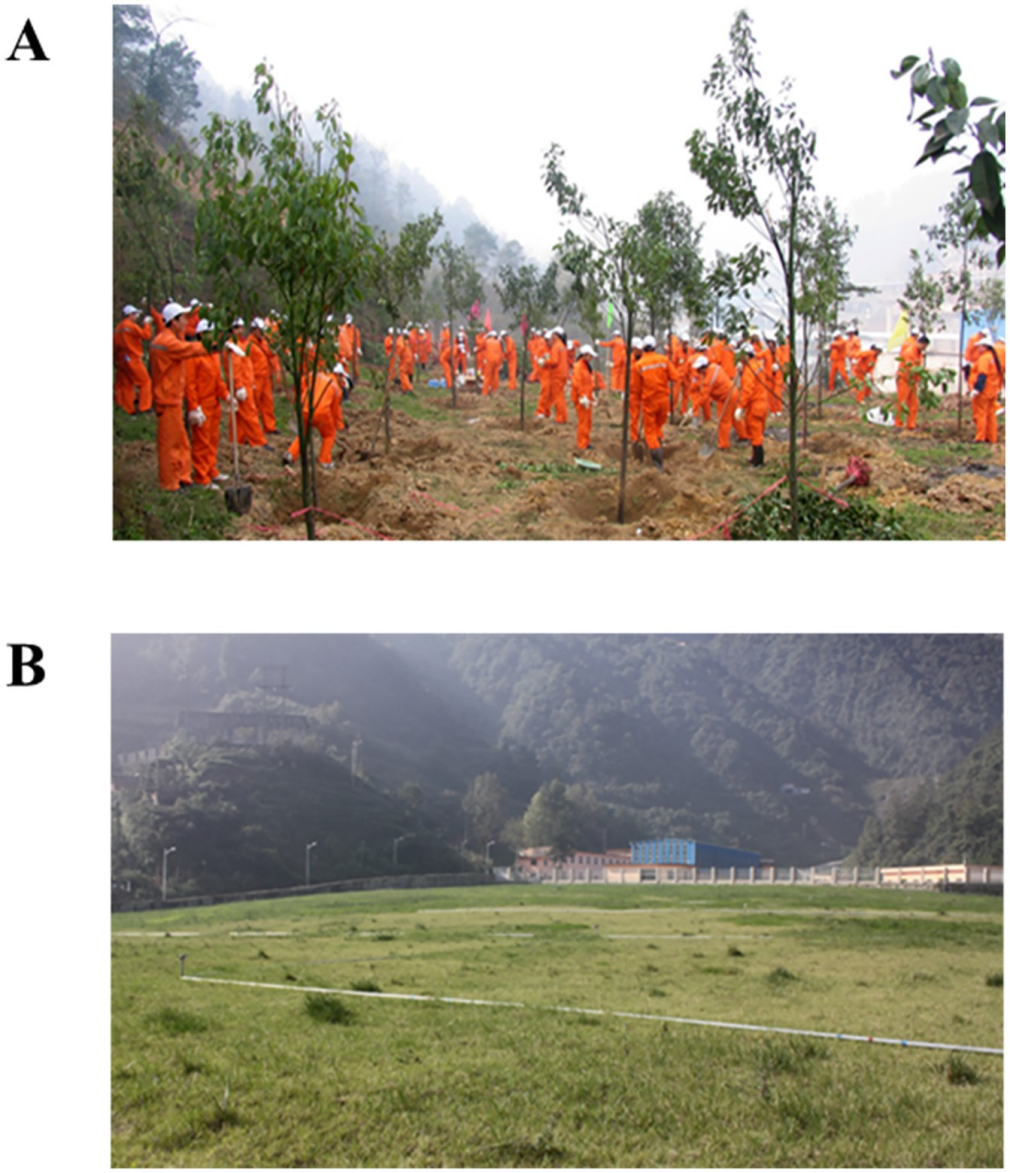
Land reclamation in mining areas. (A) Land reclamation with trees. (B) Land reclamation with grasses.

The land reclamation in Kaiyang phosphate mine is indicated in Fig 1. By using this optimal land reclamation scheme, the mutual benefits of economy and environment are attained. On the basis of the actual conditions, the proposed linguistic neutrosophic Hamacher aggregation operators can be adopted to assess the land reclamation schemes reliably and efficiently.

## 6. Discussions

In this section, the meaning of parameter *λ* in Hamacher aggregation operators is clarified by sensitivity analyses. Furthermore, the effectiveness and advantages of the proposed method are demonstrated with comparison analyses.

### 6.1 Sensitivity analyses

In subsection 5.2, when the *LNHHWAM*^*λ*^ operator with *λ = 1* is used, the ranking order *α*_1_ ≻ *α*_4_ ≻ *α*_2_ ≻ *α*_3_ is derived. In this subsection, the influence of parameter *λ* on aggregation values and ranking orders is explored with the same example.

When different *λ* values are provided in Step 4 of the proposed method, the comprehensive preference values and ranking orders with the *LNHHWAM*^*λ*^ operator and *LNHHWGM*^*λ*^ operator are itemized in Tables 5 and 6, respectively.

**Table 5:** Ranking orders with the *LNHHWAM*^*λ*^ operator under different *λ* values.

*λ*	*e*_1_	*e*_2_	*e*_3_	*e*_4_	Ranking orders	The optimal alternative
0.1	(*t*_6_, *t*_1.53_, *t*_0_)	(*t*_4.26_, *t*_0_, *t*_1.65_)	(*t*_6_, *t*_1.37_, *t*_2.00_)	(*t*_6_, *t*_1.15_, *t*_0_)	*α*_4_ ≻ *α*_1_ ≻ *α*_2_ ≻ *α*_3_	*α*_4_
0.2	(*t*_6_, *t*_1.62_, *t*_0_)	(*t*_4.24_, *t*_0_, *t*_1.56_)	(*t*_6_, *t*_1.51_, *t*_1.82_)	(*t*_6_, *t*_1.41_, *t*_0_)	*α*_4_ ≻ *α*_1_ ≻ *α*_2_ ≻ *α*_3_	*α*_4_
0.5	(*t*_6_, *t*_1.73_, *t*_0_)	(*t*_4.21_, *t*_0_, *t*_1.52_)	(*t*_6_, *t*_1.65_, *t*_1.74_)	(*t*_6_, *t*_1.67_, *t*_0_)	*α*_4_ ≻ *α*_1_ ≻ *α*_2_ ≻ *α*_3_	*α*_4_
1	(*t*_6_, *t*_1.79_, *t*_0_)	(*t*_4.18_, *t*_0_, *t*_1.52_)	(*t*_6_, *t*_1.73_, *t*_1.72_)	(*t*_6_, *t*_1.80_, *t*_0_)	*α*_1_ ≻ *α*_4_ ≻ *α*_2_ ≻ *α*_3_	*α*_1_
2	(*t*_6_, *t*_1.83_, *t*_0_)	(*t*_4.16_, *t*_0_, *t*_1.52_)	(*t*_6_, *t*_1.79_, *t*_1.72_)	(*t*_6_, *t*_1.90_, *t*_0_)	*α*_1_ ≻ *α*_4_ ≻ *α*_2_ ≻ *α*_3_	*α*_1_
5	(*t*_6_, *t*_1.87_, *t*_0_)	(*t*_4.15_, *t*_0_, *t*_1.52_)	(*t*_6_, *t*_1.84_, *t*_1.72_)	(*t*_6_, *t*_1.97_, *t*_0_)	*α*_1_ ≻ *α*_4_ ≻ *α*_2_ ≻ *α*_3_	*α*_1_
10	(*t*_6_, *t*_1.89_, *t*_0_)	(*t*_4.14_, *t*_0_, *t*_1.52_)	(*t*_6_, *t*_1.86_, *t*_1.72_)	(*t*_6_, *t*_2.00_, *t*_0_)	*α*_1_ ≻ *α*_4_ ≻ *α*_2_ ≻ *α*_3_	*α*_1_

**Table 6:** Ranking orders with the *LNHHWGM*^*λ*^ operator under different *λ* values.

*λ*	*e*_1_	*e*_2_	*e*_3_	*e*_4_	Ranking orders	The optimal alternative
0.1	(*t*_4.06_, *t*_1.99_, *t*_1.72_)	(*t*_2.33_, *t*_1.46_, *t*_1.78_)	(*t*_4.53_, *t*_2.16_, *t*_2.16_)	(*t*_4.57_, *t*_2.24_, *t*_1.58_)	*α*_4_ ≻ *α*_1_ ≻ *α*_3_ ≻ *α*_2_	*α*_4_
0.2	(*t*_4.10_, *t*_1.99_, *t*_1.70_)	(*t*_3.0.1_, *t*_1.42_, *t*_1.77_)	(*t*_4.56_, *t*_2.15_, *t*_2.15_)	(*t*_4.68_, *t*_2.22_, *t*_1.56_)	*α*_4_ ≻ *α*_1_ ≻ *α*_3_ ≻ *α*_2_	*α*_4_
0.5	(*t*_4.17_, *t*_1.97_, *t*_1.64_)	(*t*_3.68_, *t*_1.38_, *t*_1.75_)	(*t*_4.62_, *t*_2.11_, *t*_2.11_)	(*t*_4.78_, *t*_2.19_, *t*_1.52_)	*α*_4_ ≻ *α*_1_ ≻ *α*_2_ ≻ *α*_3_	*α*_4_
1	(*t*_4.25_, *t*_1.95_, *t*_1.57_)	(*t*_4.03_, *t*_1.33_, *t*_1.72_)	(*t*_4.70_, *t*_2.07_, *t*_2.07_)	(*t*_4.86_, *t*_2.15_, *t*_1.46_)	*α*_4_ ≻ *α*_2_ ≻ *α*_1_ ≻ *α*_3_	*α*_4_
2	(*t*_4.36_, *t*_1.92_, *t*_1.47_)	(*t*_4.28_, *t*_1.26_, *t*_1.29_)	(*t*_4.81_, *t*_2.02_, *t*_2.01_)	(*t*_4.95_, *t*_2.09_, *t*_1.37_)	*α*_4_ ≻ *α*_2_ ≻ *α*_1_ ≻ *α*_3_	*α*_4_
5	(*t*_4.53_, *t*_1.89_, *t*_1.30_)	(*t*_4.53_, *t*_1.15_, *t*_1.64_)	(*t*_4.99_, *t*_1.94_, *t*_1.94_)	(*t*_5.09_, *t*_2.02_, *t*_1.20_)	*α*_4_ ≻ *α*_2_ ≻ *α*_1_ ≻ *α*_3_	*α*_4_
10	(*t*_4.67_, *t*_1.87_, *t*_1.15_)	(*t*_4.69_, *t*_1.06_, *t*_1.61_)	(*t*_5.13_, *t*_1.90_, *t*_1.90_)	(*t*_5.21_, *t*_1.98_, *t*_1.05_)	*α*_4_ ≻ *α*_2_ ≻ *α*_1_ ≻ *α*_3_	*α*_4_

As shown in Table 5, it is clear that various aggregation results are obtained by the *LNHHWAM*^*λ*^ operator with diverse values of *λ*. As a result, the final ranking orders are changed when different *λ* values are chosen. Besides, it can be seen that: when 0 < *λ* < 1, the ranking result is *α*_4_ ≻ *α*_1_ ≻ *α*_2_ ≻ *α*_3_; and when *λ* ≥ 1, the ranking result is *α*_1_ ≻ *α*_4_ ≻ *α*_2_ ≻ *α*_3_.

As demonstrated in Table 6, it is true that the aggregation values by the *LNHHWGM*^*λ*^ operator with distinct *λ* values are diverse. Hence, the ultimate rankings are altered when unequal *λ* values are assigned. Moreover, it can be seen that: when 0 < *λ* < 0.5, the ranking order is *α*_4_ ≻ *α*_1_ ≻ *α*_3_ ≻ *α*_2_; when *λ* = 0.5, the ranking order is *α*_4_ ≻ *α*_1_ ≻ *α*_2_ ≻ *α*_3_; and when *λ* > 0.5, the ranking order is *α*_4_ ≻ *α*_2_ ≻ *α*_1_ ≻ *α*_3_. However, it seems that the *λ* values have no impact on the selection of the optimal alternative, because the best scheme is always *α*_4_ no matter what the value of *λ* is.

**Fig 2:**
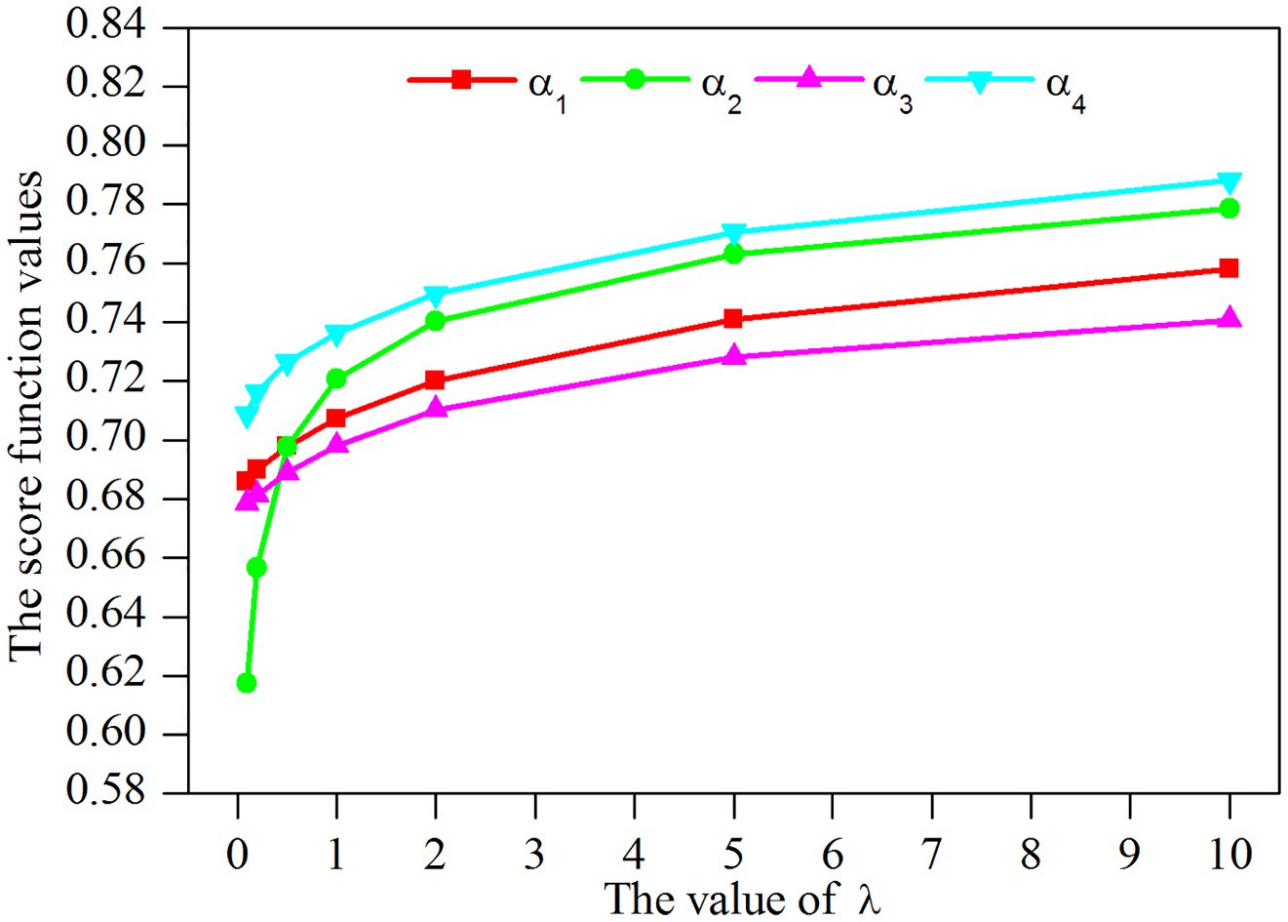
Score function values calculated by the *LNHHWAM*^*λ*^ operator.

**Fig 3:**
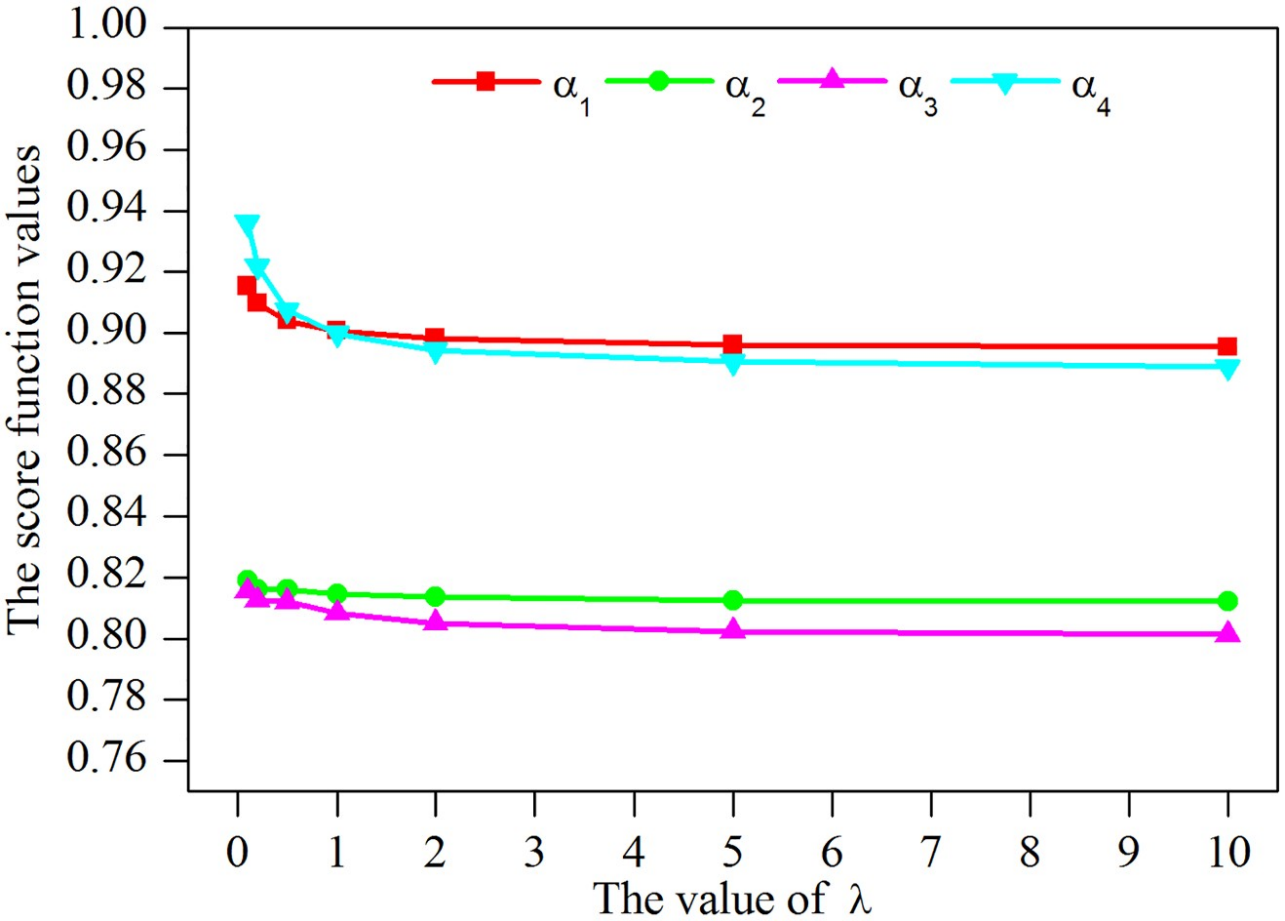
Score function values calculated by the *LNHHWGM*^*λ*^ operator.

According to Tables 5 and 6, dissimilar decisions may be made with the difference of the final rankings. To reveal the influence of parameter *λ* further, the score function values of comprehensive evaluation values calculated by the *LNHHWAM*^*λ*^ operator and *LNHHWGM*^*λ*^ operator under different *λ* values are respectively depicted in Figs 2 and 3.

As indicated in Fig 2, it can be seen that the values of score function calculated by the *LNHHWAM*^*λ*^ operator go down accordantly with the increase of *λ* values. Whereas, the values of the score function go up accordantly with the growth of *λ* values when the *LNHHWGM*^*λ*^ operator is used in Fig 3. Reasons for this phenomenon may be twofold. One is that the score function values increase as $t_{Te_{xy}}$, yet decrease as $t_{Ie_{xy}}$ and $t_{Fe_{xy}}$ in the argument values. Another important cause is that the Hamacher t-norm family monotonically rises with the intensification of *λ*, but the Hamacher t-conorm family monotonically declines with the increase of *λ* [42]. Moreover, an interesting event is that when the same values of arguments and *λ* are allocated, the values of score function calculated by the *LNHHWGM*^*λ*^ operator are invariably larger than those by the *LNHHWAM*^*λ*^ operator.

In fact, different *λ* value means dissimilar operational laws and aggregation operators. Generally, the values of the parameter *λ* = 1 or *λ* = 2 can be adopted, linguistic neutrosophic Algebraic aggregation operators or linguistic neutrosophic Einstein aggregation operators is derived in this case. Moreover, the selection of *λ* values in different aggregation operators can reflect the risk preferences of decision makers [42]. That is to say, in the *LNHHWAM*^*λ*^ operator, a big *λ* value implies the risk aversion or the pessimism of experts, and a small *λ* value indicates the risk loving or the optimism of experts. On the contrary, a completely opposite inference is drawn in the *LNHHWG0*^*λ*^ operator. Thus, specialists can determine the value of *λ* in a certain linguistic neutrosophic Hamacher aggregation operator according to their experience, risk attitudes or reality. To be specific, if the decision maker is a risk lover, *λ* = 1 with *LNHHWAM*^2^ operator and *λ* = 2 with *LNHHWGM*^2^ operator are suggested. In contrast, if the decision maker is a risk averter, *λ* = 2 with *LNHHWAM*^2^ operator and *λ* = 1 with *LNHHWGM*^1^ operator are suggested.

### 6.2 Comparison analyses

To the best of our knowledge, several major decision making methods with regard to LNNs have been suggested in literature [22–28]. In this subsection, in order to justify the validity and superiority of the proposed method, in-depth analyses are made through comparison with these existent approaches.

Part I: Theoretical comparative analyses

In this Part, the theoretical comparison results among different decision making methods in terms of numbers of parameter, computational complexity, whether capture relationships between arguments, whether consider the criteria weights objectively and whether consider the weights of ordered position are described in Table 7.

**Table 7:** Comparison results among different decision making methods.

Methods	Numbers of parameter	Computational complexity	Whether capture relationships between arguments	Whether consider the criteria weights objectively	Whether consider the weights of ordered position
Arithmetic or geometric mean operators [22]	None	Low	No	No	No
Cosine similarity [23]	None	Low	No	No	No
Extended TOPSIS [24]	None	Low	No	Yes	No
Bonferroni averaging operators [25]	Two	High	Yes	No	No
Power Heronian operators [26]	Two	High	Yes	No	No
Hamy averaging operators [27]	One	Median	Yes	Yes	No
Extended MULTIMOORA [28]	Two	High	Yes	Yes	No
The proposed method	One	Low	Yes	Yes	Yes

The detailed theoretical comparative analyses are listed as follows.

1. Compare with other aggregation operators in literature [22,25,26,27]
  First, the linguistic neutrosophic arithmetic or geometric mean operators [22] are special cases of the proposed linguistic neutrosophic Hamacher aggregation operators. Hence, the Hamacher aggregation operators are more general. In addition, the relationship between inputs can be reflected in this method. Second, the calculation of our method is relatively simpler than others [25,26,27]. Third, unlike the Bonferroni averaging operators [25] and power Heronian averaging operators [26], only one parameter needs to be determined in the proposed method. Thus, our method is more convenient and feasible.
2. Compare with classical decision making methods in literature [23,24,28]
  In general, the basic ideas of dissimilar decision making methods are different. All of them have their respective characteristics and highlights. In literature [23], the weight vector of criteria is known in advance, while an objective determination model is provided in this paper. In literature [28], the computational complexity is too high. In addition, the interrelations between arguments cannot be captured in literature [23,24]. Compared with them [23,24,28], the biggest advantage of the proposed method is that the weights of ordered positions are taken into consideration.

Part II: Numerical comparative analyses

In this Part, numerical ranking results among different decision making methods in terms of ranking orders and difference degrees are depicted in Table 8.

**Table 8:** Ranking results among different decision making methods.

Methods	Ranking orders
Arithmetic mean operators [22]	*α*_4_ ≻ *α*_1_ ≻ *α*_2_ ≻ *α*_3_
Geometric mean operators [22]	*α*_4_ ≻ *α*_2_ ≻ *α*_1_ ≻ *α*_3_
Cosine similarity [23]	*α*_4_ ≻ *α*_2_ ≻ *α*_3_ ≻ *α*_1_
Extended TOPSIS [24]	*α*_1_ ≻ *α*_2_ ≻ *α*_4_ ≻ *α*_3_
Bonferroni averaging operators [25]	*α*_1_ ≻ *α*_4_ ≻ *α*_3_ ≻ *α*_2_
Power Heronian operators [26]	*α*_4_ ≻ *α*_1_ ≻ *α*_3_ ≻ *α*_2_
Hamy averaging operators [27]	*α*_1_ ≻ *α*_3_ ≻ *α*_2_ ≻ *α*_4_
Extended MULTIMOORA [28]	*α*_1_ ≻ *α*_4_ ≻ *α*_2_ ≻ *α*_3_
The proposed method	*α*_1_ ≻ *α*_4_ ≻ *α*_2_ ≻ *α*_3_

It can be seen that dissimilar ranking orders are obtained when different decision making methods are implemented. In order to determine the optimal ranking order, an aggregation method proposed by Jahan et al. [48] is adopted as follows.

Step 1: Calculate the numbers of times a land reclamation scheme is assigned to different ranks, as shown in Table 9. Take *α*_1_ as an example, *α*_1_ has five times a ranking of 1, two times a ranking of 2 and once a ranking of 3 and 4.
Step 2: Attain the smoothing of land reclamation scheme assignment over ranks, as shown in Table 10. For each ranking, the previous column in Table 10 is added to the column of considered rank in Table 9.
Step 3: Determine the optimal ranking order. On the basis of Table 10, a linear programming model is built as Eq (68). By solving this linear programming problem, the best ranking order is determined as *α*_1_ ≻ *α*_4_ ≻ *α*_2_ ≻ *α*_3_.

$$\begin{array}{l} Max\;\Lambda =\sum_{x=1}^{4}\sum_{j=1}^{4}(\Phi_{xj}\cdot \frac{4^{2}}{j}\cdot \Gamma_{xj})\\ s.t.\{\begin{array}{c} \sum_{x=1}^{4}\Gamma_{xj}=1,\;j=1,2,3,4\\ \sum_{j=1}^{4}\Gamma_{xj}=1,\;x=1,2,3,4\\ \Gamma_{xj}=0\;or\;\Gamma_{xj}=1 \end{array} \end{array}.$$ (68)

**Table 9:** Numbers of times a land reclamation scheme is assigned to different ranks.

Schemes	Ranks			
	1	2	3	4
*α*_1_	5	2	1	1
*α*_2_		3	4	2
*α*_3_		1	3	5
*α*_4_	4	3	1	1

**Table 10:** Smoothing of land reclamation scheme assignment over ranks (Φ_*xj*_).

Schemes	Ranks			
	1	2	3	4
*α*_1_	5	7	8	9
*α*_2_	0	3	7	9
*α*_3_	0	1	4	9
*α*_4_	4	7	8	9

**Fig 4:**
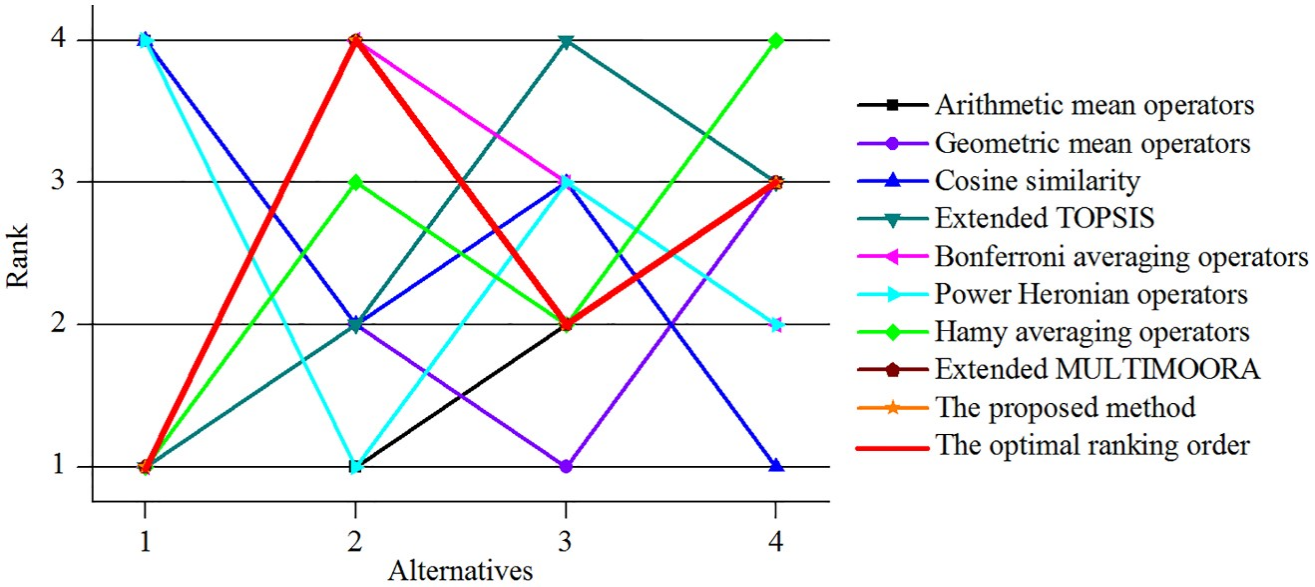
Ranking orders among different decision making methods.

For clarity, the ranking orders by using different methods are portrayed in Fig 4. It is true that the optimal ranking orders and the ranking results of the proposed approach in this paper are the same. That is to say, our method is more suitable to cope with the evaluation of land reclamation schemes for mines to some extent.

Part III: Advantages analyses

In this Part, on the basis of the analyses above, the strength of the proposed method can be summarized as follows.

1. As the Hamacher t-norm and t-conorm extend the Algebraic and Einstein t-norm and t-conorm, they have great robustness and generalization. Moreover, the new operational laws based on Hamacher t-norm and t-conorm are closed so that the logic and granularity problems can be avoided.
2. Only one parameter is contained in our method, so that the values can be readily and flexibly modified in accordance with the reality. When different parameter values are endowed, the variation of aggregation values and ranking results can be manifested dynamically. Then, the inherent rules for change can be displayed better.
3. Compared with crisp numbers and single linguistic term, the choice of LNNs is more suitable for processing complex decision making information. Furthermore, the calculation of the extended Hamacher aggregation operators for LNNs is relatively uncomplicated.
4. Not only the weights of criteria, but also the weights of ordered positions are objectively taken into account in the proposed method.

## 7. Conclusions

The evaluation of land reclamation schemes is significant for the improvement of surrounding ecological environment in mining areas. As the influence factors are numerous and intricate, the evaluation of land reclamation schemes can be deemed as a typical multi-criteria decision making problem. Considering the limitations of existed evaluation methods, a linguistic neutrosophic decision making method based on Hamacher aggregation operators was proposed to deal with such complex qualitative evaluation problem. The Hamacher operational laws of LNNs were firstly defined by utilizing the Hamacher t-norm and t-conorm. Some linguistic neutrosophic Hamacher aggregation operators were presented to aggregate linguistic neutrosophic evaluation information. Besides, important properties and special cases of these operators were discussed. The proposed operators extended the Algebraic and Einstein t-norm and t-conorm. Finally, an example was given to illustrate the effectiveness of the presented method. Meanwhile, sensitivity analyses indicated that the value of parameter may have influences on the ranking orders, and can reflect the risk attitudes of decision makers. Furthermore, the characteristics and advantages of the proposed method were verified in the comparison analyses. The largest advantage of the proposed approach is that it combined the highlights of LNNs and Hamacher aggregation operators. On the one hand, LNNs is very applicable for describing vague decision making information under complex environment. On the other hand, Hamacher aggregation operators are flexible with one parameter and have great robustness and generalization.

In the future, we plan to extend the applications of the proposed linguistic neutrosophic decision making methods, or explore more decision making methods to solve the evaluation problems of land reclamation schemes in mining areas.

## Appendix A. The proof of Theorem 1

**Proof**.

The mathematical induction is used to prove this theorem as follows.

When *n* = 1,

$$\begin{array}{ll} LNHWAM^{\lambda }(d_{1},d_{2},\cdots ,d_{n})=w_{1}d_{1}= & \\ (g^{-1}\left(\frac{{\left(1+(\lambda -1)\cdot g(t_{T_{1}})\right)}^{w_{1}}-{\left(1-g(t_{T_{1}})\right)}^{w_{1}}}{{\left(1+(\lambda -1)\cdot g(t_{T_{1}})\right)}^{w_{1}}+(\lambda -1){\left(1-g(t_{T_{1}})\right)}^{w_{1}}}\right),g^{-1}\left(\frac{\lambda {\left(g(t_{I_{1}})\right)}^{w_{1}}}{{\left(1+(\lambda -1)\left(1-g(t_{I_{1}})\right)\right)}^{w_{1}}+(\lambda -1){\left(g(t_{I_{1}})\right)}^{w_{1}}}\right),\\ g^{-1}\left(\frac{\lambda {\left(g(t_{F_{1}})\right)}^{w_{1}}}{{\left(1+(\lambda -1)\left(1-g(t_{F_{1}})\right)\right)}^{w_{1}}+(\lambda -1){\left(g(t_{F_{1}})\right)}^{w_{1}}}\right)) \end{array},$$ (69)

Eq (20) holds.

Assume when *n* = *k*, Eq (20) is true, namely,

$$\begin{array}{ll} LNHWAM^{\lambda }(d_{1},d_{2},\cdots ,d_{k})=w_{1}d_{1}⊕w_{2}d_{2}⊕\cdots ⊕w_{k}d_{k}= & \\ (g^{-1}\left(\frac{\prod_{i=1}^{k}{\left(1+(\lambda -1)\cdot g(t_{T_{i}})\right)}^{w_{i}}-\prod_{i=1}^{k}{\left(1-g(t_{T_{i}})\right)}^{w_{i}}}{\prod_{i=1}^{k}{\left(1+(\lambda -1)\cdot g(t_{T_{i}})\right)}^{w_{i}}+(\lambda -1)\prod_{i=1}^{k}{\left(1-g(t_{T_{i}})\right)}^{w_{i}}}\right),g^{-1}\left(\frac{\lambda \prod_{i=1}^{k}{\left(g(t_{I_{i}})\right)}^{w_{i}}}{\prod_{i=1}^{k}{\left(1+(\lambda -1)\left(1-g(t_{I_{i}})\right)\right)}^{w_{i}}+(\lambda -1)\prod_{i=1}^{k}{\left(g(t_{I_{i}})\right)}^{w_{i}}}\right),\\ g^{-1}\left(\frac{\lambda \prod_{i=1}^{k}{\left(g(t_{F_{i}})\right)}^{w_{i}}}{\prod_{i=1}^{k}{\left(1+(\lambda -1)\left(1-g(t_{F_{i}})\right)\right)}^{w_{i}}+(\lambda -1)\prod_{i=1}^{k}{\left(g(t_{F_{i}})\right)}^{w_{i}}}\right)) \end{array}.$$ (70)

When *n* = *k* + 1,

$$\begin{array}{l} LNHWAM^{\lambda }(d_{1},d_{2},\cdots ,d_{k+1})=(LNHWAM^{\lambda }(d_{1},d_{2},\cdots ,d_{k}))⊕w_{k+1}d_{k+1}=\\ (g^{-1}\left(\frac{\prod_{i=1}^{k}{\left(1+(\lambda -1)\cdot g(t_{T_{i}})\right)}^{w_{i}}-\prod_{i=1}^{k}{\left(1-g(t_{T_{i}})\right)}^{w_{i}}}{\prod_{i=1}^{k}{\left(1+(\lambda -1)\cdot g(t_{T_{i}})\right)}^{w_{i}}+(\lambda -1)\prod_{i=1}^{k}{\left(1-g(t_{T_{i}})\right)}^{w_{i}}}\right),g^{-1}\left(\frac{\lambda \prod_{i=1}^{k}{\left(g(t_{I_{i}})\right)}^{w_{i}}}{\prod_{i=1}^{k}{\left(1+(\lambda -1)\left(1-g(t_{I_{i}})\right)\right)}^{w_{i}}+(\lambda -1)\prod_{i=1}^{k}{\left(g(t_{I_{i}})\right)}^{w_{i}}}\right),\\ g^{-1}\left(\frac{\lambda \prod_{i=1}^{n}{\left(g(t_{F_{i}})\right)}^{w_{i}}}{\prod_{i=1}^{k}{\left(1+(\lambda -1)\left(1-g(t_{F_{i}})\right)\right)}^{w_{i}}+(\lambda -1)\prod_{i=1}^{k}{\left(g(t_{F_{i}})\right)}^{w_{i}}}\right))⊕(g^{-1}\left(\frac{{\left(1+(\lambda -1)\cdot g(t_{T_{k+1}})\right)}^{w_{k+1}}-{\left(1-g(t_{T_{k+1}})\right)}^{w_{k+1}}}{{\left(1+(\lambda -1)\cdot g(t_{T_{k+1}})\right)}^{w_{k+1}}+(\lambda -1){\left(1-g(t_{T_{k+1}})\right)}^{w_{k+1}}}\right),\\ g^{-1}\left(\frac{\lambda {\left(g(t_{I_{k+1}})\right)}^{w_{k+1}}}{{\left(1+(\lambda -1)\left(1-g(t_{I_{k+1}})\right)\right)}^{w_{k+1}}+(\lambda -1){\left(g(t_{I_{k+1}})\right)}^{w_{k+1}}}\right),g^{-1}\left(\frac{\lambda {\left(g(t_{F_{k+1}})\right)}^{w_{k+1}}}{{\left(1+(\lambda -1)\left(1-g(t_{F_{k+1}})\right)\right)}^{w_{k+1}}+(\lambda -1){\left(g(t_{F_{k+1}})\right)}^{w_{k+1}}}\right)) \end{array}$$ (71)

Let $A_{i}={\left(1+(\lambda -1)(g(t_{T_{i}}))\right)}^{w_{i}}$, $B_{i}={\left(1-g(t_{T_{i}})\right)}^{w_{i}}$, $C_{i}={\left(1+(\lambda -1)(1-g(t_{I_{i}}))\right)}^{w_{i}}$, $D_{i}={\left(g(t_{I_{i}})\right)}^{w_{i}}$, $E_{i}={\left(1+(\lambda -1)(1-g(t_{F_{i}}))\right)}^{w_{i}}$ and $P_{i}={\left(g(t_{F_{i}})\right)}^{w_{i}}$ for all *i* = 1,2,…,*k*, then

$$\begin{array}{l} LNHWAM^{\lambda }(d_{1},d_{2},\cdots ,d_{k+1})=\\ \left(g^{-1}\left(\frac{\prod_{i=1}^{k}A_{i}-\prod_{i=1}^{k}B_{i}}{\prod_{i=1}^{k}A_{i}+(\lambda -1)\prod_{i=1}^{k}B_{i}}\right),g^{-1}\left(\frac{\lambda \prod_{i=1}^{k}D_{i}}{\prod_{i=1}^{k}C_{i}+(\lambda -1)\prod_{i=1}^{k}D_{i}}\right),g^{-1}\left(\frac{\lambda \prod_{i=1}^{n}P_{i}}{\prod_{i=1}^{k}E_{i}+(\lambda -1)\prod_{i=1}^{k}P_{i}}\right)\right)⊕\\ \left(g^{-1}\left(\frac{A_{k+1}-B_{k+1}}{A_{k+1}+B_{k+1}}\right),g^{-1}\left(\frac{\lambda D_{k+1}}{C_{k+1}+(\lambda -1)D_{k+1}}\right),g^{-1}\left(\frac{\lambda P_{k+1}}{E_{k+1}+(\lambda -1)P_{k+1}}\right)\right)=\\ (g^{-1}\left(\frac{\left(\prod_{i=1}^{k}A_{i}-\prod_{i=1}^{k}B_{i})\left(\prod_{i=1}^{k}A_{i}+(\lambda -1)\prod_{i=1}^{k}B_{i}\right)+(A_{k+1}-B_{k+1})\left(\prod_{i=1}^{k}A_{i}+(\lambda -1)\prod_{i=1}^{k}B_{i}\right)-(\prod_{i=1}^{k}A_{i}-\prod_{i=1}^{k}B_{i})(A_{k+1}-B_{k+1})-(1-\lambda )(\prod_{i=1}^{k}A_{i}-\prod_{i=1}^{k}B_{i})(A_{k+1}-B_{k+1}\right)}{\left(\prod_{i=1}^{k}A_{i}+(\lambda -1)\prod_{i=1}^{k}B_{i}\right)\left(A_{k+1}+(\lambda -1)B_{k+1}\right)-(1-\lambda )(\prod_{i=1}^{k}A_{i}-\prod_{i=1}^{k}B_{i})(A_{k+1}-B_{k+1})}\right),\\ g^{-1}\left(\frac{\lambda^{2}\prod_{i=1}^{k+1}D_{i}}{\lambda \left(\prod_{i=1}^{k}C_{i}+(\lambda -1)\prod_{i=1}^{k}D_{i}\right)\left(C_{k+1}+(\lambda -1)D_{k+1}\right)+(1-\lambda )\left(\prod_{i=1}^{k}\lambda D_{i}(C_{k+1}+(\lambda -1)D_{k+1}\right)+\lambda D_{k+1}\left(\prod_{i=1}^{k}C_{i}+(\lambda -1)\prod_{i=1}^{k}D_{i}\right)-\lambda^{2}\prod_{i=1}^{k+1}D_{i})}\right),\\ g^{-1}\left(\frac{\lambda^{2}\prod_{i=1}^{k+1}P_{i}}{\lambda \left(\prod_{i=1}^{k}E_{i}+(\lambda -1)\prod_{i=1}^{k}P_{i}\right)\left(E_{k+1}+(\lambda -1)P_{k+1}\right)+(1-\lambda )\left(\prod_{i=1}^{k}\lambda P_{i}(E_{k+1}+(\lambda -1)P_{k+1}\right)+\lambda P_{k+1}\left(\prod_{i=1}^{k}E_{i}+(\lambda -1)\prod_{i=1}^{k}P_{i}\right)-\lambda^{2}\prod_{i=1}^{k+1}P_{i})}\right))\\ =\left(g^{-1}\left(\frac{\prod_{i=1}^{k}A_{i}-\prod_{i=1}^{k}B_{i}}{\prod_{i=1}^{k}A_{i}+(\lambda -1)\prod_{i=1}^{k}B_{i}}\right),g^{-1}\left(\frac{\lambda \prod_{i=1}^{k}D_{i}}{\prod_{i=1}^{k}C_{i}+(\lambda -1)\prod_{i=1}^{k}D_{i}}\right),g^{-1}\left(\frac{\lambda \prod_{i=1}^{n}P_{i}}{\prod_{i=1}^{k}E_{i}+(\lambda -1)\prod_{i=1}^{k}P_{i}}\right)\right)⊕\\ \left(g^{-1}\left(\frac{A_{k+1}-B_{k+1}}{A_{k+1}+B_{k+1}}\right),g^{-1}\left(\frac{\lambda D_{k+1}}{C_{k+1}+(\lambda -1)D_{k+1}}\right),g^{-1}\left(\frac{\lambda P_{k+1}}{E_{k+1}+(\lambda -1)P_{k+1}}\right)\right)\\ =\left(g^{-1}\left(\frac{\lambda (\prod_{i=1}^{k+1}A_{i}-\prod_{i=1}^{k+1}B_{i})}{\lambda \prod_{i=1}^{k+1}A_{i}+\lambda (\lambda -1)\prod_{i=1}^{k+1}B_{i}}\right),g^{-1}\left(\frac{\lambda^{2}\prod_{i=1}^{k+1}D_{i}}{\lambda \prod_{i=1}^{k+1}C_{i}+\lambda (\lambda -1)\prod_{i=1}^{k+1}D_{i}}\right),g^{-1}\left(\frac{\lambda^{2}\prod_{i=1}^{k+1}P_{i}}{\lambda \prod_{i=1}^{k+1}E_{i}+\lambda (\lambda -1)\prod_{i=1}^{k+1}P_{i}}\right)\right)\\ =\left(g^{-1}\left(\frac{\prod_{i=1}^{k+1}A_{i}-\prod_{i=1}^{k+1}B_{i}}{\prod_{i=1}^{k+1}A_{i}+(\lambda -1)\prod_{i=1}^{k+1}B_{i}}\right),g^{-1}\left(\frac{\lambda \prod_{i=1}^{k+1}D_{i}}{\prod_{i=1}^{k+1}C_{i}+(\lambda -1)\prod_{i=1}^{k+1}D_{i}}\right),g^{-1}\left(\frac{\lambda \prod_{i=1}^{k+1}P_{i}}{\prod_{i=1}^{k+1}E_{i}+(\lambda -1)\prod_{i=1}^{k+1}P_{i}}\right)\right) \end{array}$$ (72)

Therefore,

$$\begin{array}{l} LNHWAM^{\lambda }(d_{1},d_{2},\cdots ,d_{k})=\\ (g^{-1}\left(\frac{\prod_{i=1}^{k+1}{\left(1+(\lambda -1)\cdot g(t_{T_{i}})\right)}^{w_{i}}-\prod_{i=1}^{k+1}{\left(1-g(t_{T_{i}})\right)}^{w_{i}}}{\prod_{i=1}^{k+1}{\left(1+(\lambda -1)\cdot g(t_{T_{i}})\right)}^{w_{i}}+(\lambda -1)\prod_{i=1}^{k+1}{\left(1-g(t_{T_{i}})\right)}^{w_{i}}}\right),g^{-1}\left(\frac{\lambda \prod_{i=1}^{k+1}{\left(g(t_{I_{i}})\right)}^{w_{i}}}{\prod_{i=1}^{k+1}{\left(1+(\lambda -1)\left(1-g(t_{I_{i}})\right)\right)}^{w_{i}}+(\lambda -1)\prod_{i=1}^{k+1}{\left(g(t_{I_{i}})\right)}^{w_{i}}}\right),\\ g^{-1}\left(\frac{\lambda \prod_{i=1}^{k+1}{\left(g(t_{F_{i}})\right)}^{w_{i}}}{\prod_{i=1}^{k+1}{\left(1+(\lambda -1)\left(1-g(t_{F_{i}})\right)\right)}^{w_{i}}+(\lambda -1)\prod_{i=1}^{k+1}{\left(g(t_{F_{i}})\right)}^{w_{i}}}\right)) \end{array},$$ (73)

and Eq (20) is true.

On the other hand, since $\frac{\prod_{i=1}^{k+1}{\left(1+(\lambda -1)\cdot g(t_{T_{i}})\right)}^{w_{i}}-\prod_{i=1}^{k+1}{\left(1-g(t_{T_{i}})\right)}^{w_{i}}}{\prod_{i=1}^{k+1}{\left(1+(\lambda -1)\cdot g(t_{T_{i}})\right)}^{w_{i}}+(\lambda -1)\prod_{i=1}^{k+1}{\left(1-g(t_{T_{i}})\right)}^{w_{i}}}\in [0,1]$, $\frac{\lambda \prod_{i=1}^{k+1}{\left(g(t_{I_{i}})\right)}^{w_{i}}}{\prod_{i=1}^{k+1}{\left(1+(\lambda -1)\left(1-g(t_{I_{i}})\right)\right)}^{w_{i}}+(\lambda -1)\prod_{i=1}^{k+1}{\left(g(t_{I_{i}})\right)}^{w_{i}}}\in [0,1]$ and $\frac{\lambda \prod_{i=1}^{k+1}{\left(g(t_{F_{i}})\right)}^{w_{i}}}{\prod_{i=1}^{k+1}{\left(1+(\lambda -1)\left(1-g(t_{F_{i}})\right)\right)}^{w_{i}}+(\lambda -1)\prod_{i=1}^{k+1}{\left(g(t_{F_{i}})\right)}^{w_{i}}}\in [0,1]$, then the aggregated results using Eq (20) is still a LNN.

Now, Theorem 1 is proved.

## References

[1] Ali SH, Giurco D, Arndt N, Nickless E, Brown G, Demetriades A, et al. Mineral supply for sustainable development requires resource governance. Nature 2017; 543(7645): 367–372. doi: 10.1038/nature21359 28300094

[2] Wood BJ. Mineral resources and the limits to growth. Elements 2017; 13(5): 291–292.

[3] Calas G. Mineral resources and sustainable development. Elements 2017; 13(5): 301–306.

[4] Palogos I, Galetakis M, Roumpos C, Pavloudakis F. Selection of optimal land uses for the reclamation of surface mines by using evolutionary algorithms. International Journal of Mining Science and Technology 2017; 27(3): 491–498.

[5] Swab RM, Lorenz N, Byrd S, Dick R. Native vegetation in reclamation: Improving habitat and ecosystem function through using prairie species in mine land reclamation. Ecological Engineering 2017; 108: 525–536.

[6] Bascetin A. A decision support system using analytical hierarchy process (AHP) for the optimal environmental reclamation of an open-pit mine. Environmental Geology 2007; 52(4): 663–672.

[7] Pavloudakis F, Galetakis M, Roumpos C. A spatial decision support system for the optimal environmental reclamation of open-pit coal mines in Greece. International Journal of Mining, Reclamation and Environment 2009; 23(4): 291–303.

[8] Soltanmohammadi H, Osanloo M, Bazzazi AA. An analytical approach with a reliable logic and a ranking policy for post-mining land-use determination. Land Use Policy 2010; 27(2): 364–372.

[9] Wang SD, Liu CH, Zhang HB. Suitability evaluation for land reclamation in mining area: A case study of Gaoqiao bauxite mine. Transactions of Nonferrous Metals Society of China 2011; 21: S506–S515.

[10] Cheng LL, Hu ZQ, Lou S. Improved methods for fuzzy comprehensive evaluation of the reclamation suitability of abandoned mine lands. International Journal of Mining, Reclamation and Environment 2017; 31(3): 212–229.

[11] Liu PD, Li DF. Some muirhead mean operators for intuitionistic fuzzy numbers and their applications to group decision making. PloS one 2017; 12(1): e0168767. doi: 10.1371/journal.pone.0168767 28103244 PMC5245779

[12] Zadeh LA. Fuzzy sets. Information and Control 1965; 8(3): 338–353.

[13] Ma LH, Chen H, Yan HZ, Yang LF, Wu LF. Multiple attribute decision making model and application to food safety risk evaluation. PloS one 2017; 12(12): e0189835. doi: 10.1371/journal.pone.0189835 29261754 PMC5736234

[14] Liang WZ, Zhao GY, Luo SZ. Selecting the optimal mine ventilation system via a decision making framework under hesitant linguistic environment. Symmetry 2018; 10(7): 283.

[15] Tiwari AK, Shreevastava S, Som T, Shukla KK. Tolerance-based intuitionistic fuzzy-rough set approach for attribute reduction. Expert Systems with Applications 2018; 101: 205–212.

[16] Luo SZ, Zhang HY, Wang JQ, Li L. Group decision-making approach for evaluating the sustainability of constructed wetlands with probabilistic linguistic preference relations. Journal of the Operational Research Society 2018; 10.1080/01605682.2018.1510806

[17] Garg H, Nancy. Non-linear programming method for multi-criteria decision making problems under interval neutrosophic set environment. Applied Intelligence 2018; 48(8): 2199–2213.

[18] Zhou W, Yin WY, Peng XQ, Liu FM, Yang F. Comprehensive evaluation of land reclamation and utilisation schemes based on a modified VIKOR method for surface mines. International Journal of Mining, Reclamation and Environment 2018; 32(2): 93–108.

[19] Xu ZS. Deviation measures of linguistic preference relations in group decision making. Omega 2005; 33(3): 249–254.

[20] Luo SZ, Cheng PF, Wang JQ, Huang YJ. Selecting project delivery systems based on simplified neutrosophic linguistic preference relations. Symmetry 2017; 9(8): 151.

[21] Zhang ST, Zhu JJ, Liu XD, Chen Y, Ma ZZ. Adaptive consensus model with multiplicative linguistic preferences based on fuzzy information granulation. Applied Soft Computing 2017; 60: 30–47.

[22] Fang ZB, Ye J. Multiple attribute group decision-making method based on linguistic neutrosophic numbers. Symmetry 2017; 9(7): 111.

[23] Shi LL, Ye J. Cosine measures of linguistic neutrosophic numbers and their application in multiple attribute group decision-making. Information 2017; 8(4): 117.

[24] Liang WZ, Zhao GY, Wu H. Evaluating investment risks of metallic mines using an extended TOPSIS method with linguistic neutrosophic numbers. Symmetry 2017; 9(8): 149.

[25] Fan CX, Ye J, Hu KL, Fan E. Bonferroni mean operators of linguistic neutrosophic numbers and their multiple attribute group decision-making methods. Information 2017; 8(3): 107.

[26] Liu PD, Mahmood T, Khan Q. Group decision making based on power Heronian aggregation operators under linguistic neutrosophic environment. International Journal of Fuzzy Systems 2018; 20(3): 970–985.

[27] Liu PD, You XL. Some linguistic neutrosophic Hamy mean operators and their application to multi-attribute group decision making. PloS one 2018; 13(3): e0193027. doi: 10.1371/journal.pone.0193027 29513697 PMC5841783

[28] Liang WZ, Zhao GY, Hong CS. Selecting the optimal mining method with extended multi-objective optimization by ratio analysis plus the full multiplicative form (MULTIMOORA) approach. Neural Computing and Applications 2018; 10.1007/s00521-018-3405-5

[29] Nancy, Garg H. Novel single-valued neutrosophic decision making operators under Frank norm operations and its application. International Journal for Uncertainty Quantification 2016; 6(4): 361–375.

[30] Garg H, Nancy. Some hybrid weighted aggregation operators under neutrosophic set environment and their applications to multicriteria decision-making. Applied Intelligence 2018; 10.1007/s10489-018-1244-9

[31] Liu PD, Qin XY. Maclaurin symmetric mean operators of linguistic intuitionistic fuzzy numbers and their application to multiple-attribute decision-making. Journal of Experimental & Theoretical Artificial Intelligence 2017; 29(6): 1173–1202.

[32] Garg H, Nancy. Linguistic single-valued neutrosophic prioritized aggregation operators and their applications to multiple-attribute group decision-making. Journal of Ambient Intelligence & Humanized Computing 2018; 10.1007/s12652-018-0723-5

[33] Garg H, Kumar K. Some aggregation operators for linguistic intuitionistic fuzzy set and its application to group decision-making process using the set pair analysis. Arabian Journal for Science and Engineering 2017; 43(6): 3213–3227.

[34] Garg H, Nancy. Multi-criteria decision-making method based on prioritized Muirhead mean aggregation operator under neutrosophic set environment. Symmetry 2018; 10(7): 280.

[35] Liu PD, Liu JL, Merigó JM. Partitioned Heronian means based on linguistic intuitionistic fuzzy numbers for dealing with multi-attribute group decision making. Applied Soft Computing 2018; 62: 395–422.

[36] Oussalah M. On the use of Hamacher’s t-norms family for information aggregation. Information Sciences 2003; 153: 107–154.

[37] Yan HB, Huynh VN, Nakamori Y, Murai T. On prioritized weighted aggregation in multi-criteria decision making. Expert Systems with Applications 2011; 38(1): 812–823.

[38] Liu PD. Some Hamacher aggregation operators based on the interval-valued intuitionistic fuzzy numbers and their application to group decision making. IEEE Transactions on Fuzzy systems 2014; 22(1): 83–97.

[39] Garg H. Some series of intuitionistic fuzzy interactive averaging aggregation operators. Springerplus 2016; 5: 999. doi: 10.1186/s40064-016-2591-9 27441128 PMC4937014

[40] Garg H, Agarwal N, Tripathi A. Some improved interactive aggregation operators under interval-valued intuitionistic fuzzy environment and their application to decision making process. Scientia Iranica 2017; 24(5): 2581–2604.

[41] Gao H, Wei GW, Huang YH. Dual hesitant bipolar fuzzy Hamacher prioritized aggregation operators in multiple attribute decision making. IEEE Access 2018; 6: 11508–11522.

[42] Wu Q, Wu P, Zhou LG., Chen HY, Guan XJ. Some new Hamacher aggregation operators under single-valued neutrosophic 2-tuple linguistic environment and their applications to multi-attribute group decision making. Computers & Industrial Engineering 2017; 116: 144–162.

[43] Li BL, Wang JR, Yang LH, Li XT. Multiple criteria decision making approach with multivalued neutrosophic linguistic normalized weighted Bonferroni mean Hamacher operator. Mathematical Problems in Engineering 2018; 10.1155/2018/2432167

[44] Tang J, Meng FY. Linguistic intuitionistic fuzzy Hamacher aggregation operators and their application to group decision making. Granular Computing 2018; 10.1007/s41066-018-0089-2

[45] Liang WZ, Zhao GY, Hong CS. Performance assessment of circular economy for phosphorus chemical firms based on VIKOR-QUALIFLEX method. Journal of Cleaner Production 2018; 196: 1365–1378.

[46] Wang WZ, Liu XW. Intuitionistic fuzzy geometric aggregation operators based on Einstein operations. International Journal of Intelligent Systems 2011; 26(11): 1049–1075.

[47] Liang WZ, Zhao GY, Wu H, Chen Y. Assessing the risk degree of goafs by employing hybrid TODIM method under uncertainty. Bulletin of Engineering Geology and the Environment 2018; 10.1007/s10064-018-1340-4

[48] Jahan A, Ismail MY, Shuib S, Norfazidah D, Edwards KL. An aggregation technique for optimal decision-making in materials selection. Materials & Design 2011; 32(10): 4918–4924.

